# Analysis of Family Structures Reveals Robustness or Sensitivity of Bursting Activity to Parameter Variations in a Half-Center Oscillator (HCO) Model

**DOI:** 10.1523/ENEURO.0015-16.2016

**Published:** 2016-08-30

**Authors:** Anca Doloc-Mihu, Ronald L. Calabrese

**Affiliations:** Department of Biology, Emory University, Atlanta, Georgia 30322

**Keywords:** analysis, computation, database, half-center oscillator, invertebrates, robustness

## Abstract

The underlying mechanisms that support robustness in neuronal networks are as yet unknown. However, recent studies provide evidence that neuronal networks are robust to natural variations, modulation, and environmental perturbations of parameters, such as maximal conductances of intrinsic membrane and synaptic currents. Here we sought a method for assessing robustness, which might easily be applied to large brute-force databases of model instances. Starting with groups of instances with appropriate activity (e.g., tonic spiking), our method classifies instances into much smaller subgroups, called families, in which all members vary only by the one parameter that defines the family. By analyzing the structures of families, we developed measures of robustness for activity type. Then, we applied these measures to our previously developed model database, HCO-db, of a two-neuron half-center oscillator (HCO), a neuronal microcircuit from the leech heartbeat central pattern generator where the appropriate activity type is alternating bursting. In HCO-db, the maximal conductances of five intrinsic and two synaptic currents were varied over eight values (leak reversal potential also varied, five values). We focused on how variations of particular conductance parameters maintain normal alternating bursting activity while still allowing for functional modulation of period and spike frequency. We explored the trade-off between robustness of activity type and desirable change in activity characteristics when intrinsic conductances are altered and identified the hyperpolarization-activated (h) current as an ideal target for modulation. We also identified ensembles of model instances that closely approximate physiological activity and can be used in future modeling studies.

## Visual Abstract

**Figure:**
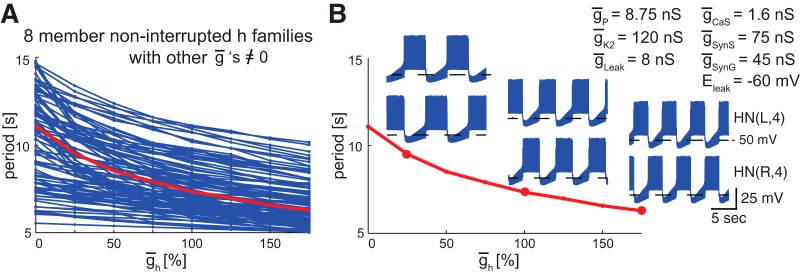

## Significance Statement

Robustness is an attribute of living systems and mathematical models that describe them. We developed a method for assessing the robustness of activity types (e.g., bursting), which can be applied to brute-force databases of neuronal model instances in which biologically relevant parameters are varied, and where sensitivity analyses are conceptually and practically difficult to apply. By organizing all instances with appropriate activity into families, in which all members vary only by the one parameter defining the family, we developed measures of robustness for activity type based on family structure and address a fundamental challenge to robustness, modulation, which, by changing parameters, may alter activity type. The method determines which parameters predictably alter activity characteristics, (e.g., burst period), without changing activity type.

## Introduction

Robustness is a fundamental feature of complex systems (Kitano, 2004) like neuronal networks, yet remains only operationally defined, and the underlying mechanisms that support it are largely unknown (Gutierrez et al., 2013; Marder et al., 2015). If robustness for a neuronal network is defined as the maintenance of a desirable activity state in the face of parameter variation (e.g., maximal conductance of intrinsic membrane and synaptic currents), then abundant experimental evidence (Swensen and Bean, 2005; Schultheiss et al., 2012; Talebi and Baker, 2012; Tang et al., 2012; Langen et al., 2013; Caplan et al., 2014; Dethier et al., 2015;) suggests that neuronal networks are robust to animal-to-animal variations, modulation, and environmental perturbations of these parameters.

Modulation poses a particular challenge to network robustness, because it often must modify network activity without changing activity type (e.g., changing the period of a rhythmically active network without disrupting its rhythmicity or phase). Some modeling studies have addressed the mechanisms underlying such functional robustness (Goldman et al., 2001, 2003; O’Leary et al., 2014). Also, sensitivity analysis is commonly used to assess the influence of a parameter on activity characteristics and type in neuronal models (Olypher and Calabrese, 2007), but is difficult to apply to multidimensional parameter spaces that neurons occupy, though there has been some notable progress (Marder et al., 2014; Drion et al., 2015).

The purpose of this study was twofold, with a focus on assessing robustness. First, we developed a strategy for assessing the robustness of model neurons applicable to large databases of model instances based on a grid structure (brute-force). We classified instances in the database into small groups called families in which members vary only by the value of the parameter that defines the family, and we used the family structures to develop new measures for assessing the robustness (or sensitivity) of electrical activity to changes in model parameters. Second, we applied our measures to an existing model of a two-neuron half-center oscillator (HCO).

Central pattern generators (CPGs; Marder, 2011; Calabrese, 2014) pace adaptable rhythmic behaviors, such as walking and breathing. Their inherent rhythmicity and adaptability to perturbations results from the interaction of their intrinsic and synaptic properties with neuromodulatory and sensory inputs. CPGs exhibit remarkably robust activity types (e.g., rhythmic alternating bursting), yet extensive modulation of activity characteristics (e.g., burst period) occurs. Thus, they represent an excellent test bed for exploring the interplay of robustness and modifiability.

Reciprocally inhibitory neurons (often autonomous bursters) called HCOs are prevalent circuit building blocks of CPGs that assure robust alternating bursting (Selverston et al., 2000; Cymbalyuk et al., 2002). HCO models display a wide range of bursting activity when intrinsic and synaptic conductances of the neurons are varied (Prinz et al., 2004). We used the HCO model of Hill et al., 2001', which reproduces the electrical activity observed in the leech heartbeat CPG and consists of a pair of reciprocally inhibitory model neurons represented as single isopotential compartments with intrinsic and synaptic conductances of the type defined by Hodgkin and Huxley (1952). Each model neuron contains eight voltage-dependent currents, five inward currents (*I*_Na_, a fast Na^+^ current; *I*_P_, a persistent Na^+^ current; *I*_CaF_, rapidly inactivating low-threshold Ca current; *I*_CaS_, slowly inactivating low-threshold Ca current; and *I*_h_, a hyperpolarization-activated cation current), three outward currents (*I*_K1_, a delayed rectifier-like K current; *I*_K2_, a persistent K current; and *I*_KA_, a fast transient K current), and two types of inhibitory synaptic transmission: graded (*I*_SynG_) and spike mediated (*I*_SynS_).

To explore the HCO parameter space [maximal conductances (ḡ values) of intrinsic and synaptic currents], we simulated ∼10.5 million model instances, whose characteristics we recorded into a database named HCO-db (Doloc-Mihu and Calabrese, 2011). Here, we systematically explored the parameter space of two identified groups from the database: realistic HCOs or (*r*HCOs), which show normal physiological activity with 99,066 instances; and functional HCOs (*f*HCOs), which show nonphysiological but functional alternating bursting activity with 1.1 million instances. We subdivided instances from both groups into families of instances that vary by only one parameter that defines the family. By examining family structures and patterns, we developed new measures of robustness of bursting activity to alterations of model parameters. Using this analysis, we showed that the HCO model is robust to variations of *I*_h_ but is highly sensitive to the *I*_P_. Moreover, the burst period is reliably and predictably regulated by modulating ḡ_h_, suggesting that it is ideal for neuromodulation. This new analysis also allowed us to identify ensembles of instances that show typical robust physiological activity for future analysis of parameter variations.

## Materials and Methods

### Half-center oscillator (HCO) model

We used a half-center oscillator model (Hill et al., 2001) that produces rhythmic alternating bursting activity resembling the electrical activity in the heartbeat CPG of the leech. This model is publicly available on ModelDB repository (https://senselab.med.yale.edu/ModelDB; accession #19698). The model consists of a two reciprocally inhibitory model interneurons, represented as single isopotential electrical compartments with intrinsic and synaptic membrane conductances of the Hodgkin and Huxley (1952) type. Each model neuron contains, in addition to a leakage current, eight voltage-dependent currents, five inward currents (*I*_Na_, *I*_P_, *I*_CaF_, *I*_CaS_, and *I*_h_), and three outward currents (*I*_K1_, *I*_K2_, and *I*_KA_). In what follows, this half-center oscillator model is simply referred to as the model or the model neurons, and the currents are referred to by their letters. The model has two types of inhibitory synaptic transmission between the two interneurons: SynG and SynS. The graded transmission SynG was modeled as a postsynaptic conductance controlled by presynaptic Ca^2+^ concentration and the spike-mediated transmission SynS was modeled as a postsynaptic conductance triggered by presynaptic spikes. The values for the maximal conductances and the leak reversal potential are the free parameters in the model. For our canonical model, these values are ḡ_CaS_ = 3.2 nS, ḡ_h_ = 4 nS, ḡ_P_ = 7 nS, ḡ_K2_ = 80 nS, ḡ_Leak_ = 8 nS, ḡ_SynS_ = 60 nS, ḡ_SynG_ = 30 nS, ḡ_Na_ = 200 nS, ḡ_CaF_ = 5 nS, ḡ_K1_ = 100 nS, ḡ_KA_ = 80 nS, and E_leak_ = −60 mV (Doloc-Mihu and Calabrese, 2011). The kinetics, voltage dependencies, reversal potentials of the intrinsic currents, and the synaptic connections of the HCO model interneurons have all been verified and previously adjusted to fit the biological data of leech interneurons (Hill et al., 2001). The differential equations of the model are given in the study by Doloc-Mihu and Calabrese, 2014.

In previous work, Doloc-Mihu and Calabrese, 2011 performed extensive simulations of this model by systematically varying eight key parameters (a brute-force approach). All model simulations were started from the same initial conditions, which were different for each of the two neurons and were obtained by running the canonical HCO model (Hill et al., 2001) for 200 s, such that one of the two neurons was in its bursting state and the other one was being inhibited. The same parameter values were used in each of the paired model neurons. The eight parameters varied were as follows: seven maximal conductances (ḡ_SynS_, ḡ_SynG_, ḡ_Leak_, ḡ_P_, ḡ_CaS_, ḡ_h_, and ḡ_K2_), across 0%, 25%, 50%, 75%, 100%, 125%, 150%, and 175% of their canonical values, and E_leak_ across −70, −65, −60, −55, and −50 mV. After changing a parameter, we ran each model instance for 100 s to allow the system to establish stable activity, and then we ran it for another 100 s, from which we recorded the voltage traces of the electrical activity corresponding to its paired neurons and the corresponding spike times. The firing characteristics were analyzed and recorded in a database. By systematically varying the eight key parameters (a brute-force approach) in all possible combinations, we developed a database of 10,485,760 simulated model instances named “HCO-db” (available upon request; see description in Doloc-Mihu and Calabrese, 2011). The resulting parameter space includes 10,321,920 instances, which have at least one synaptic component present and thus are potential working HCOs, and 163,840 isolated neuron instances, which contain twin neurons without any synaptic interaction.

### Definitions

In voltage traces, we recognized a spike only if the potential waveform crossed a threshold of −20 mV. We defined a burst as having at least three spikes and a minimum interburst interval of 1 s. We defined the burst period as being the interval between the middle spikes of two consecutive bursts. Phase was calculated on a per burst basis, as being the delay from the middle spike of a burst of neuron B to the middle spike of the preceding burst of neuron A divided by the interval from this middle spike of the next burst of neuron A to the middle spike of the preceding burst of neuron A. The duty cycle was defined as the percentage of the period occupied by a burst. Each burst spike frequency is defined as the number of spikes within the burst divided by the burst duration. The spike frequency of a neuron is defined as the mean of all burst spike frequencies divided by the mean of all burst durations.

We defined a half-center oscillator instance (here referred to simply as HCO) as having two model interneurons each showing bursting activity with at least two bursts in a 40 s time interval, with each burst having normal spikes (coefficient of variation of the amplitudes of the spikes within any burst, <0.07); a small variation of period (coefficient of variation of period, < 0.05); relative phase in the range of 0.45–0.55; and at least one synaptic component present (either ḡ_SynS_ ≠ 0, or ḡ_SynG_ ≠ 0, or both ḡ_SynS_ ≠ 0 and ḡ_SynG_ ≠ 0). We considered an *r*HCO instance as being an HCO that showed physiological bursting corresponding to that observed in leech oscillator heart interneurons. Precisely, it was an HCO with period between 5 and 15 s, average spike frequency between 8 and 25 Hz, and duty cycle between 50% and 70%. All HCO instances that did not meet the criteria to be designated as realistic (i.e., *r*HCOs) were designated as *f*HCOs because they still maintained functional alternating bursting. Note that *r*HCOs and *f*HCOs are nonoverlapping subsets of HCOs.

We defined an isolated neuron instance (isolated neuron) as having two identical interneurons (though started with different initial conditions, but otherwise identical), and no synaptic interaction (i.e., ḡ_SynS_ = 0 and ḡ_SynG_ = 0). We defined a burster instance as being an isolated neuron instance for which both neurons had at least two bursts, each with normal spikes, and regular periods (as defined above for the HCOs). We defined a realistic burster as being a burster that showed realistic bursting corresponding to isolated leech oscillator heart interneurons. Precisely, it was a burster with a period between 5 and 15 s, and an average spike frequency between 8 and 25 Hz. Note that realistic bursters can be thought of as being HCOs with no synaptic connections.

We define a family as being a subset (of a group) of instances that all have the same parameter values except the one that defines the family (e.g., all realistic bursters that vary only by ḡ_P_ constitute a family of P). We define a family sequence as the order of values (e.g., increasing) of its defining parameter. Note that each family member has a unique ḡ value of the defining parameter, and all the ḡ values within the same family form the family sequence. A family sequence is delimited by a beginning and an ending parameter value (permissible range), not by the grid limits of the database. If the family sequence includes all grid points in the database within the permissible range, this family is called “noninterrupted”; if not all grid points within the permissible range are members of the family sequence, then the family is called “interrupted.” In the latter case, we say that the family sequence has broken down.

By using our definitions from above as criteria, we identified from our HCO-db database those four groups that include simulated instances showing appropriate burst characteristics (period, spike frequency, duty cycle): 99,066 *r*HCOs (realistic); 1,103,073 *f*HCOs (functional not realistic); 307 realistic bursters; and 117 bursters that are not realistic. By querying our HCO-db database, we analyzed (with our own Java and Matlab scripts) the sensitivity of the leech burst characteristics to changes in maximal conductances for the realistic groups of instances.

### Sensitivity classification

For each family, plotting the burst characteristics (period, spike frequency) versus the ḡ value of each family member and then connecting the points obtained corresponding to the adjacent family members via lines forms a curve. We built and used our own Matlab scripts to automatically separate the families according to their curve steepness calculated as the maximum slope. As a family curve is not smooth but is made of several connected line segments, it was important first to assess its monotonicity. Then, the algorithm can be applied easily. The algorithm used the polyfit and polyval functions in Matlab (code inspired from Matlab Central, Jean-Luc Dellis “getthetangent”: http://www.mathworks.com/matlabcentral/fileexchange/23799-getthetangent/content/getthetangent.m) to fit a smooth curve (polynomial of degree 5 was enough for most h families) to our family curves. The slope of a curve at a certain point is the slope of the tangent at that point. So, the algorithm finds the tangent to the curve with the maximum slope (calculated by using the derivative of the curve). The tangent to a family curve gave the slope or the angle of the decay (with respect to the *x*-axis). Our algorithm first found the families that decline steeply and classified them as the high-sensitivity group; then it found the families that had a gradual decline and classified them as the low-sensitivity group; and finally the rest of the families were classified as the medium-sensitivity group. We set up a threshold of −2.5 for the high-sensitivity group (angle range, ∼68–90^°^ clockwise), and a maximum of −0.4 for the low-sensitivity group (angle range, ∼0–22° clockwise).

## Results

We started our study by first searching for mechanisms involving correlated conductance parameters (ḡ values) that influence the robustness of activity type, here realistic HCO bursting activity, since parameter correlation is one mechanism that produces and maintains robustness. Recent work (Doloc-Mihu and Calabrese, 2014) investigated the potential relationships between parameters that maintain bursting activity in isolated neurons from the HCO model (bursters, see Definitions, in Materials and Methods). The authors found a linearly correlated set of three maximal conductances (of leak current, ḡ_Leak_; of a persistent K current, ḡ_K2_; and of a persistent Na^+^ current, ḡ_P_) that maintains bursting activity in burster (including realistic burster) model instances, therefore underlying the robustness of bursting activity. In addition, they found that bursting activity was very sensitive to individual variation of these parameters; only correlated changes could maintain the activity type. Now we ask whether or not there is a similar linear correlation between maximal conductances of the ḡ_Leak_ of the ḡ_K2_ and of the ḡ_P_ that maintains realistic HCO bursting activity.

### Nonlinear correlation between ḡ_P,_ ḡ_Leak,_ ḡ_K2,_ and E_leak_ for realistic HCOs

**Figure 1.**
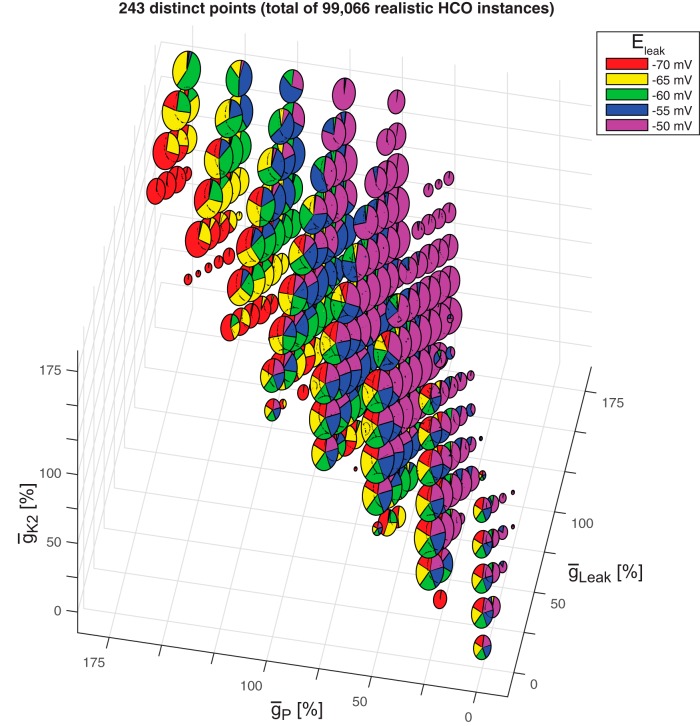
3D view of the 99,066 realistic HCO instances. Plot of all *r*HCO instances projected onto the 3D space given by the maximal conductances of *I*_P_, *I*_K2_, and *I*_Leak._ Each point displays a pie chart of the E_leak_ of all instances from the group having the same values of ¯g_P_, ¯g_K2_, and ¯g_Leak_ as the respective projected point. The number of instances projected onto each point in the 3D space is shown by the size of the E_leak_ pie (as radius). For a better visualization of the points, we applied the natural logarithm to the sizes of the pies.

To address this question, we developed a Matlab script to visualize the following five characteristics of a dataset at once: three parameters, which form a 3D parameter space of the data (here, *r*HCOs), the number of instances projected onto each point in this space given by the size of each point, and pie charts of a fourth parameter showing all instances projected onto each point in the 3D space. Here, the three parameters forming the 3D space were ḡ_P_, ḡ_K2_, and ḡ_Leak_, and the fourth parameter was E_leak_. The plot obtained is shown in Figure 1. We selected these parameters inspired by the research in the study by Doloc-Mihu and Calabrese, 2014. The pie chart was split into five slices according to the number of values possible for E_leak_, with each slice having a different color. If there was no instance projected into the 3D space for a particular value of the fourth parameter, then its corresponding slice was not shown in the pie chart. For a better visualization of the points projected onto the 3D plot (Fig. 1), we used the natural logarithm to size the pies (as radius). For example, the largest point from the plot included 2,440 projected realistic HCO instances, with 488 per each E_leak_ slice, and its pie size was ln(2440) = 7.7998.

The plot in Figure 1 shows that there is no linear correlation among ḡ_P_, ḡ_K2_, and ḡ_Leak_ that is similar to the one observed for bursters in the study by Doloc-Mihu and Calabrese, 2014, which showed a clustering of the points along the main diagonal. The 3D shape from Figure 1 has a complicated contour, similar to a wedge, and reveals potential nonlinear correlation among these three maximal conductances. A similar wedge-like shape correlation was observed by Goldman et al. (2001, their Fig. 2) among maximal conductances of A, Ca, Na, and KCa currents in a theoretical study on lobster superior temporal gyrus neurons.

**Figure 2.**
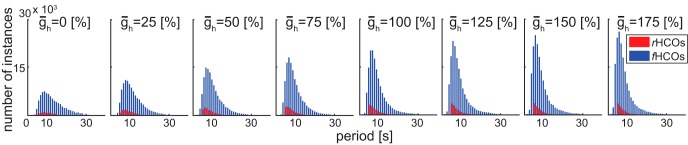
The number of instances within the *r*HCO group and *f*HCO group vs their period values for each value of ¯g_h_. *f*HCOs are in blue and *r*HCOs are in red. Period values were between 1 and 40 s for the *f*HCOs and between 5 and 15 s for the *r*HCOs. Plots were obtained using the bar Matlab function (R2014a). Each bar shows the period values within one unit or between [*n*, *n* + 1). The bars corresponding to the realistic instances were shifted with 0.3 on the *x*-axis for better visualization. ¯g_h_ values are given as the percentage of the canonical (100%).

Expansions of all the E_leak_ pie charts revealed that the main diagonal is a border between E_leak_ values: above the main diagonal were predominantly more positive values of E_leak_ (−50 and −55 mV); along the diagonal were all E_leak_ values; and below the diagonal were mostly more negative values (−70 and −65 mV). The number of instances seemed to be biased toward the main diagonal as the biggest pies can be seen along it. That is, realistic HCO activity is influenced (produced) by almost equal (including ±25% and ±50% variations) values of ḡ_P_, ḡ_K2_, and ḡ_Leak_ (range, 0–175%), along with middle values of E_leak_ (−55, −60, and −65 mV).

However, while a linear correlation among ḡ_P_, ḡ_K2_, and ḡ_Leak_ was not observed for the realistic HCO instances, these three parameters (along with E_leak_) defined a limited parameter space in which realistic activity was observed. Now we wanted to ask how other parameters (working individually) influence the alternating bursting activity in the HCOs, given the constraints imposed by E_leak_, ḡ_Leak_, ḡ_K2_, and ḡ_P_.

### Increasing ḡ_h_ promotes bursting

Here, we answer this question by first looking at how varying the value of ḡ_h_ affects bursting activity in HCOs and realistic HCOs. From now on, we separate the HCOs into two nonoverlapping groups, the *r*HCOs and *f*HCOs, which include all HCOs that are not realistic HCOs.

Many *f*HCOs (461,724 instances) had only the duty cycle outside the physiological range [0.5–0.7] for the three criteria used to determine realistic status. Also, there were many (155,556 instances) *f*HCOs that had only spike frequencies that were too high (>25 Hz) or too low (<8 Hz). There were relatively fewer *f*HCOs (23,523 instances) that had burst periods that were too low (<5 s) or too high (>15 s). A good number of *f*HCOs (84,586 instances) did not satisfy any of the physiological criteria. Also, there were *f*HCOs that did not meet two of these three realistic requirements, in every combination: 30,751 *f*HCOs failed period and spike frequency; 87,025 *f*HCOs failed period and duty cycle; and 259,908 *f*HCOs failed spike frequency and duty cycle.

Figure 2 illustrates the number of instances within each of the *r*HCO and *f*HCO groups versus with their period values for each value of ḡ_h_. Figure 2 reveals right-skewed distributions of the *f*HCO and *r*HCO instances for each h value. For *f*HCOs, increasing the value of ḡ_h_ has the following two effects: it monotonically increases the number of instances within the group (from 94,639 instances for ḡ_h_ = 0 to 181,083 for ḡ_h_ = 175%), and it increases the number of instances with faster bursting activity (periods between 5 and 10 s). For the latter, we used the skewness Matlab function to show that the *f*HCOs distributions have positive monotonically increasing skewness values (from 0.95 for ḡ_h_ = 0 to 1.93 for ḡ_h_ = 175%) as the value of ḡ_h_ increases. For *r*HCOs, Figure 2 shows a peak in the number of these instances for ḡ_h_ = 75%. Similar to the distributions of the *f*HCOs, the distributions of the *r*HCOs show positive monotonically increasing skewness values (from 1.38 for ḡ_h_ = 0 to 2.9 for ḡ_h_ = 175%) as the value of ḡ_h_ increases. We conclude that a larger ḡ_h_ value promotes functional and realistic HCO bursting.

The amount of h current thus appears to be important in regulating alternating bursting in mutually inhibitory heart interneurons. Moreover, increasing ḡ_h_ values increases the number of instances with shorter burst periods (between 5 and 10 s). Next, we investigated in more detail the effect of each parameter on the realistic bursting activity.

### Robustness as defined by the family size

For each parameter, we queried the HCO-db database to build up the families (of instances) existing for that particular parameter within the *r*HCO group (we also build up the families within *f*HCOs; data not shown). Then, we partitioned the *r*HCOs into families according to the number of members in the family (see Definitions). Table 1 summarizes, for each parameter, the number of families of each size (number of members) within the *r*HCOs. From Table 1, we can see that P has many families (on the order of thousands) with a small number of members (one and two members) and no large families (with three to eight members). K2 shows a family structure similar to that of P, with thousands of families with a small number of members (one to three members), but also has a small number of families with four and five members. Leak, CaS, and SynS share a similar family structure, with thousands of families with a small number of members (one to three members), and a good number of families with four to six members. Finally, h and SynG show a different family structure, including a large number of families with seven and eight members, and thousands of the rest.

**Table 1:** The number of families for each parameter within the realistic HCO group

	One member	Two members	Three members	Four members	Five members	Six members	Seven members	Eight members
P	92,970	3,048 (3,048)						
K2	68,702	13,163 (11,836)	1,299 (1,223)	34 (34)	1 (1)			
Leak	66,611	11,873 (10,305)	2,133 (1,569)	452 (301)	92 (54)	7 (6)		
CaS	43,500	17,851 (13,471)	5,263 (3,854)	929 (765)	67 (63)	4 (4)		
SynS	38,588	16,950 (11,131)	6,390 (3,341)	1,542 (832)	236 (121)	10 (7)		
h	16,877	9,895 (5,545)	6,325 (2,525)	4,322 (1,411)	2,703 (930)	1,285 (415)	537 (240)	144 (144)
SynG	10,551	7,047 (2,830)	5,086 (1,276)	4,124 (865)	3,046 (535)	1,971 (483)	1,061 (448)	1,023 (1,023)

Families with the same number of members are grouped together. In parentheses, we show the number of noninterrupted families.

### Family sequence

In this work, we used the ḡ values within the same family in increasing order of their values to form the family sequence (see Definitions). By analyzing the family sequences, we revealed that many families have internal gaps or breaks. A family sequence is delimited by a beginning and an end parameter value (permissible range), not by the grid limits of the database. A family is noninterrupted if its sequence includes all grid points in the database between these limits; otherwise, the family is called interrupted (i.e., if there is at least one missing grid point from the family sequence between these limits). In Table 1, we present in parentheses the number of noninterrupted families. The percentage of noninterrupted families within the total number of families of a given number of members varied for different parameters. For ḡ_K2_, it showed a steady increase with increasing number of family members from 89.9% to 100%. For ḡ_P_, we had all families as noninterrupted families (100%). For the rest of the parameters, the percentage of noninterrupted families fluctuated (i.e., changed nonmonotonically), as follows: between 58.7% and 86.7% for ḡ_Leak_ (corresponding to families with five and two members, respectively); between 73.2% and 100% for ḡ_CaS_ (for families with three and six members, respectively); between 51.2% and 70% for ḡ_SynS_ (for families with five and six members, respectively); between 32.3% and 100% for ḡ_h_ (for families with six and eight members, respectively); and between 17.5% and 100% for ḡ_SynG_ (for families with five and eight members, respectively). Interestingly, both ḡ_h_ and ḡ_SynG_ showed a similar tendency: both had a large number of families with any given number of members, and, with the exception of their families with eight members, both displayed very low numbers of noninterrupted families (<56% for ḡ_h_ and <42.2% for ḡ_SynG_).

Here, we used a large number of families with many members (best if all eight members are present) as a measure of the robustness of realistic bursting activity over the defining parameter changing within these families. Moreover, noninterrupted families indicated strong robustness to the variation of the defining family parameter. Using this criterion, from above we can conclude that realistic HCO activity is robust to changes in h and SynG, and that it is sensitive to changes in P and maybe K2. Next, we looked at the family beginning and end values of the defining ḡ, for each ḡ value, along with their potential sequence interruptions to assess potential robustness or sensitivity.

### Robustness as defined by family sequence breakdown

For each parameter, we plotted the distributions of its families that were interrupted. The presence of interruptions in a family sequence indicates that realistic HCO activity is not robust over the entire permissible range of the family’s defining parameter, with all other parameters remaining constant. Conversely, noninterrupted (or continuous) families indicate robustness to the variation of the defining family parameter.

#### Sequence breakdown versus changes in ḡ_h_

**Figure 3.**
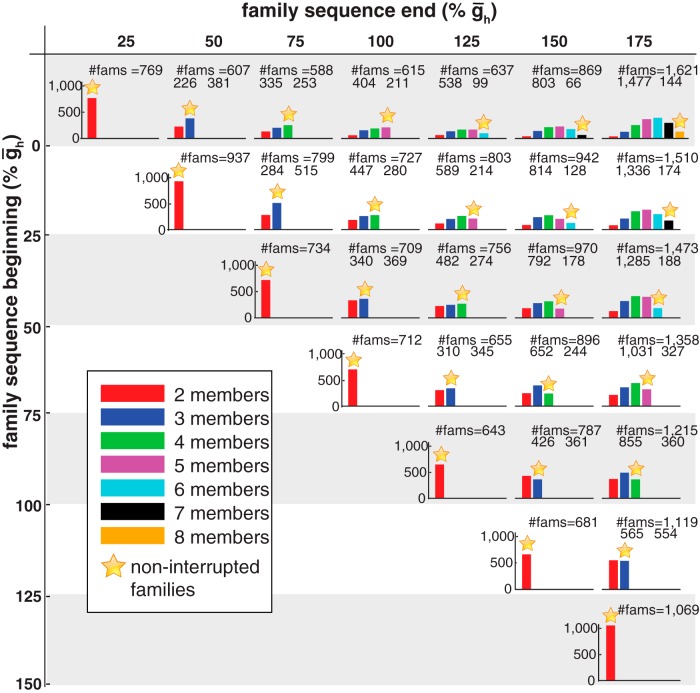
Distribution of h families of *r*HCOs based on the sequence breakdown of their members. Labels at the margins indicate the beginning (vertical dimension) and end (horizontal dimension)¯g_h_ values of the family sequences. Colors indicate the number of members within the family. The titles of panels show the total number of families [total number of interrupted families plus the number of noninterrupted families (star)]. Numbers under each panel title give the total number of interrupted families (left) and the number of noninterrupted families (right). For each panel, the *y*-axis indicates the number of families. Bins corresponding to the families having all members with noninterrupted̄g_h_ values are marked with an orange star. All panels show data at the same scale.

Figure 3 illustrates the distributions of interrupted and noninterrupted h families for the *r*HCOs. There were a total of 25,057 families with two to seven members, of which 11,066 were noninterrupted and 13,991 were interrupted. As the permissible range of the family sequence increased—move along any given row (beginning point) toward the right-most column (largest end point)—the number of interrupted families increases, while the number of noninterrupted families tends to decrease then increase for the largest sequence end point (ḡ_h_ = 175%). For large permissible ranges, there are a large number of families with multiple interruptions in their sequences. Conversely, the smaller the permissible range, the larger the proportion of noninterrupted families. This analysis also points out that with a large permissible range (>4) of ḡ_h_, regardless of the beginning or end point permissible, there are a considerable number of families with a sizeable number of members (more than four), albeit some with interruptions, and there are many families that have such permissible ranges (4,669). What is somewhat surprising about these results is that there are so many interruptions in family sequences, and for large permissible ranges there can be several interruptions. It is thus important to explore whether these interruptions lead to a change in activity type or reflect a more subtle change like a movement beyond the physiological range criteria (e.g., spike frequency) for an *r*HCO, while maintaining *f*HCO activity.

#### Sequence breakdown versus changes in ḡ_CaS_

**Figure 4.**
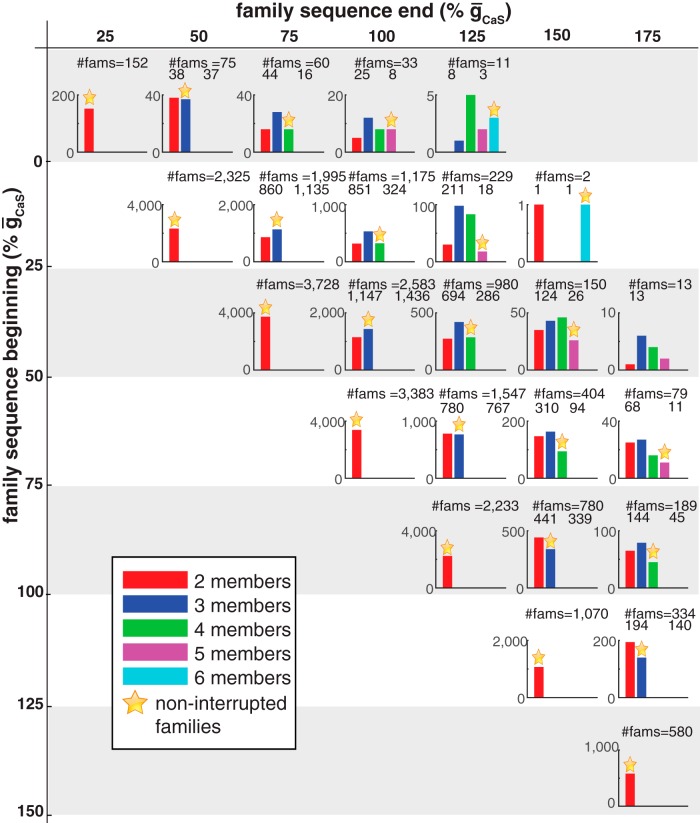
Distribution of CaS families of realistic HCOs based on the sequence breakdown of their members. Labels at the margins indicate the beginning (vertical dimension) and end (horizontal dimension)¯g_CaS_ values of the family sequences. Colors indicate the number of members within the family. The titles of panels show the total number of families [total number of interrupted families plus the number of noninterrupted families (star)]. The numbers under each panel title give the total number of interrupted families (left) and the number of noninterrupted families (right). For each panel, the *y*-axis indicates the number of families. Bins corresponding to the families having all members with noninterrupted̄g_CaS_ values are marked with an orange star. Empty panels have been removed. To visualize bins with few families, panels are at different scales.

Figure 4 illustrates the distributions of interrupted and noninterrupted CaS families for the *r*HCOs. There were a total of 24,110 CaS families with two to six members, of which 18,157 were noninterrupted and 5,953 were interrupted. No family is thus able to span the entire range of ḡ_CaS_ values tested. Figure 4 shows that the number of interrupted families generally decreases as the permissible range increases (look across rows), which is in stark contrast to h families (Fig. 3), except for sequences that start at ḡ_CaS_ = 0, where there is a peak for sequences that end at ḡ_CaS_ = 75%. Similarly, the number of noninterrupted families decreases as the permissible range increases (look across rows). This analysis points out that there are no families with seven or eight members and few with six members (4 families) with a modest number of families with five members (67 families), most of which are noninterrupted (63 families). Still, as with h families, there can be several interruptions in family sequences except for families with permissible ranges of approximately four. As with h families, it is important to explore whether these interruptions lead to a change in activity type or reflect a more subtle change like a movement beyond the physiological range criteria (e.g., spike frequency) for an *r*HCO, while maintaining *f*HCO activity.

#### Sequence breakdown versus changes in ḡ_P_

For *r*HCOs, we obtained only families with two members for P, and all these families are noninterrupted (Table 1). There were no families of P that started at 0. The peak in the number of P families was obtained for families starting at 50% and ending at 75% (1,614 families), followed by families starting at 75% and ending at 100% (861 families).

#### Sequence breakdown versus changes in ḡ_K2_

For *r*HCOs, there were a total of 14,497 K2 families with two to five members, of which 13,144 families were noninterrupted. The number of K2 families, both noninterrupted and interrupted, increased monotonically as the end point of the sequence increased, with the largest number (4,600 and 741, respectively) of these families for ḡ_K2_ = 175%. There was an increase in the number of families as the ḡ_K2_ end point increased, which suggests that more ḡ_K2_ promotes realistic HCO bursting.

#### Sequence breakdown versus changes in ḡ_Leak_

For *r*HCOs, there were a total of 14,557 leak families with two to six members, of which 12,273 were noninterrupted. The maximum number of noninterrupted families of any size (number of members) was when the sequence started with no leak present (ḡ_Leak_ = 0), and as the amount of ḡ_Leak_ increased the number of noninterrupted families decreased monotonically. Lower ḡ_Leak_ values (0–100%) seemed to promote more noninterrupted families and, thus, resulted in more robust realistic HCO activity.

#### Sequence breakdown versus changes in ḡ_SynS_

There were a total of 25,128 SynS families with two to six members, of which 15,432 were noninterrupted. For each number of members, the number of noninterrupted families increased monotonically and steeply as the sequence beginning point increased and also as the sequence end point increased (peak at 175%). These observations suggest that stronger spike-mediated synaptic transmission promoted robust realistic HCO activity.

#### Sequence breakdown versus changes in ḡ_SynG_

There were a total of 22,335 SynG families with two to seven members, of which 6,437 were noninterrupted. Like h, SynG had many families (1,023 families) with all eight possible members (noninterrupted sequence). As the sequence beginning point increased (peak at 0), both the number of noninterrupted families and interrupted families decreased monotonically and steeply (from 1,904 and 8,267 for ḡ_SynG_ = 0 respectively, to 384 and 218, respectively, for ḡ_SynG_ = 150% and ḡ_SynG_ = 125%). So, it appears that more ḡ_SynG_ did not promote robustness.

### Robustness as shown by missing family members

In the following, we analyze the missing members from all *r*HCO families (noninterrupted and interrupted); the missing members outside the family’s permissible range (beginning and end points); and the missing members in the family sequence to determine the proportion of missing members that maintain functional HCO activity (regular alternating bursting but not realistic). We use this as a measure of the robustness of alternating bursting activity state.

#### Bursting activity shows robustness to changes in ḡ_h_

**Figure 5.**
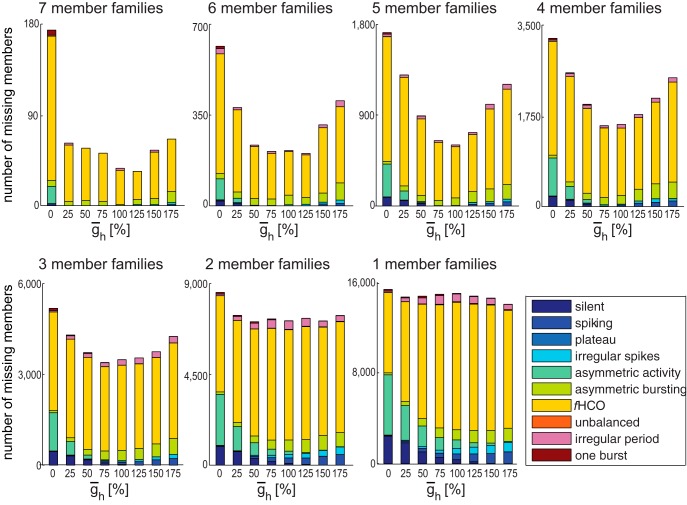
Distribution of missing members from h families of *r*HCOs for each̄g_h_ value. The activity type of the missing members is color coded as shown in the legend.

The plots in Figure 5 illustrate the distributions of the missing members from h families of the *r*HCOs for each  ḡ_h_ value. In these plots, the bars at ḡ_h_ = 0% and ḡ_h_ = 175% values contain only missing members outside the family’s permissible range, and all bars but these extremes are mixed. As family size decreases, more of the missing values illustrated are from beyond the family’s permissible range. We grouped together all families with the same number of members (and thus the same number of missing members) and showed their data on the same subplot. We color coded the activity type of the missing members. It is easy to see that yellow, the color corresponding to the *f*HCOs, is predominant, which reveals that most (from 85% for families with seven members to 68% for families with one member) of the missing members from the interrupted h families of the *r*HCOs are in fact *f*HCO instances. Table 2 shows the number of missing (h) family members of *r*HCOs that have functional HCO bursting grouped by the physiological criterion or criteria that they fail. For all families, regardless of size (number of members), the biggest number of missing instances from *r*HCO interrupted families did not meet the duty cycle criterion. A very small number of these missing instances from *r*HCO families did not satisfy any of the three realistic criteria. In addition, Table 1 shows that there was a large number of multimember h families of the *r*HCOs (with more than a third of them being noninterrupted), including 144 perfect h families with all eight members present (circuit stability/strength). These observations show that variations in ḡ_h_ might move HCO bursting outside the physiological range but still keep it in the functional HCO range, which shows the robustness of the functional alternating bursting activity to these variations.

**Table 2:** The number of missing h family members of *r*HCOs that have functional HCO bursting classified according to the physiological criteria that they fail

	Period	Spike frequency	Duty cycle	Period and spike frequency and duty cycle	Period and spike frequency	Period and duty cycle	Spike frequency and duty cycle
Two members	3,759	3,679	26,449	51	496	3,691	4,324
Three members	2,188	1,597	16,102	31	288	1,923	1,716
Four members	1,286	598	9,538	22	154	1,087	633
Five members	656	195	4,643	22	96	559	216
Six members	162	57	1,628	0	3	146	37
Seven members	31	9	393	0	0	24	2

#### Bursting activity shows robustness to changes in ḡ_CaS_, if it is sufficiently high

Distributions (data not shown) of the missing members from CaS families of the *r*HCOs for each ḡ_CaS_ value revealed that, if the model neurons had low ḡ_CaS_ values (0, 25%, and 50%), most of the missing members from the CaS families (∼61%) had continuous spiking activity, and that if the model neurons had sufficiently high ḡ_CaS_ (at least 75%), then most (between 79% for families with one member and 95.6% for families with five members) of the missing members from these families had *f*HCO bursting activity. That is, a minimum of ḡ_CaS_ = 75% was the cutoff for system robustness; changes in ḡ_CaS_ values <75% will result in changes in the activity type, and changes in ḡ_CaS_ values starting from 75% will result in keeping the functional HCO bursting activity.

#### Bursting activity shows sensitivity (no robustness) to changes in ḡ_P_

**Figure 6.**
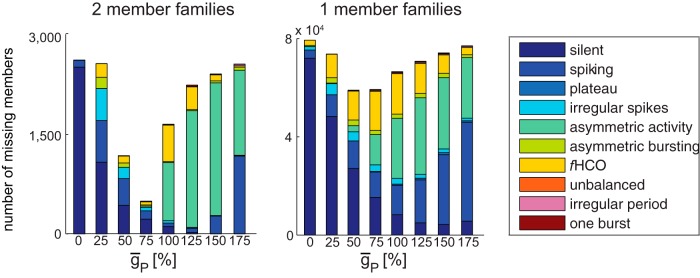
Distribution of missing members from P families of *r*HCOs for each ¯g_P_ value. The activity type of the missing members is color coded as shown in the legend.

Table 1 shows that the *r*HCOs had P families with only one or two members, and none of these are interrupted. The plots in Figure 6 show the distributions of the missing members from interrupted P families of the *r*HCOs for each ḡ_P_ value; thus, the plots in Figure 6 had seven and six missing members, respectively. These plots reveal that the activity of the *r*HCOs was quite sensitive to changes in the ḡ_P_ value: any change in ḡ_P_ value was very likely (>85.6%) to move the activity type outside functional HCO bursting. For ḡ_P_ values <100%, the activity type became predominantly silent (60%) or spiking (12%), and for ḡ_P_ values ≥100% the bursting activity became (between 28.8% for families with one member and 66.7% for families with two members) asymmetric (e.g., one cell is silent while the other cell is spiking; Doloc-Mihu and Calabrese, 2011), continuous spiking (between 25.8% for families with one member and 17.4% for families with two members), functional (13.6%), or silent (8%, but only for families with one member). In summary, the activity became predominantly nonfunctional (i.e., no alternating bursting).

#### Bursting activity shows more sensitivity (than robustness) to changes in ḡ_K2_

Table 1 shows that K2 had mainly noninterrupted families (13,094), with some interrupted families (1,403). The distributions of the missing members from K2 families for the *r*HCOs (data not shown) reveal that *r*HCO activity was quite sensitive to changes in ḡ_K2_ value: a change in ḡ_K2_ value was very likely (>81%) to move the activity type outside functional HCO bursting. For low ḡ_K2_ values (0% or 25%), the activity type became mainly spiking or plateau, and for the rest of the ḡ_K2_ values the activity became mostly asymmetric, to a lesser extent spiking, or in a significant number of cases *f*HCO (varying from 16% to 26% across ḡ_K2_ values), which was very similar to the sensitivity to changes in P current. Increasing the amount of ḡ_K2_ changed the activity type from spiking to bursting, but mostly asymmetric bursting and not *f*HCO activity.

#### Bursting activity shows moderate sensitivity to changes in ḡ_Leak_

Table 1 shows that Leak had mostly noninterrupted families (12,235) with a fairly large proportion of interrupted families (2,322). Distributions of the missing members from Leak families for the *r*HCOs (data not shown) reveal that the activity of the *r*HCOs was quite sensitive to changes in ḡ_Leak_ values, as the proportion of missing members that showed functional HCO activity was low, varying between 21% and 51% across ḡ_Leak_ values. Increasing ḡ_Leak_ values >100% increased the proportion of missing members with spiking activity and decreased the proportion of missing members with asymmetric activity (i.e., outside the *f*HCO activity). It seems that ḡ_Leak_ = 100% was the cutoff for model sensitivity: any ḡ_Leak_ value above it decreased the proportion of missing family members with *f*HCO bursting. This observation indicates that high ḡ_Leak_ values move the model neurons outside the *f*HCO bursting range, but lower ḡ_Leak_ values can maintain activity inside the *f*HCO bursting.

#### Bursting activity shows robustness to changes in ḡ_SynS_ towards higher values

Table 1 shows that SynS had many noninterrupted families (15,432) but also many interrupted families (9,696). Distributions of the missing members from SynS families for the *r*HCOs (data not shown) revealed that low values of ḡ_SynS_ (<100%) produced mostly (between 57.7% and 93.7% across ḡ_SynS_ values) missing members with spiking activity, and that for sufficient values of ḡ_SynS_ (≥100%) most (between 64.5% and 96.8%) missing members of the SynS families had *f*HCO bursting activity. As the amount of ḡ_SynS_ increased, there were substantially more missing members with *f*HCO activity and fewer missing members with spiking activity within each group of families with the same number of missing members. This means that a strong spike-mediated synapse was necessary (at least ḡ_SynS_ = 100%) to maintain *f*HCO bursting activity. In addition, the relatively large number of multimember SynS families of the *r*HCOs shows that *r*HCO bursting activity was quite robust to variations of the spike-mediated synapse (ḡ_SynS_); variations of ḡ_SynS_, especially toward large values (at least ḡ_SynS_ = 100%) maintained either realistic or functional HCO bursting activity.

#### Bursting activity shows robustness to changes in ḡ_SynG_

Table 1 shows that SynG had mainly interrupted families (15,898) with a significant proportion of noninterrupted families (6,437). Distributions of the missing members from SynG families for the *r*HCOs (data not shown) revealed that most (between 75% and 81%) of the missing members from the SynG families of the *r*HCOs were in fact *f*HCO instances. This observation indicates that varying ḡ_SynG_ does not disrupt *f*HCO bursting activity. Based also on the large number of multimember SynG families of the *r*HCOs, including 1,023 families with all eight members present, it seems that in the HCO model realistic bursting activity was robust to variations of the ḡ_SynG_; variations in ḡ_SynG_ values maintained either realistic or functional HCO bursting activity of the neurons.

### Sensitivity of burst characteristics to variations of ḡ

To assess the sensitivity of a characteristic (period, spike frequency) to the variation of a parameter, we plotted the burst characteristic (period or spike frequency) versus the corresponding maximal conductance values of all family members for each of the families (of the parameter considered) that have the same number of members. Then, we connected via a line the burst characteristic values corresponding to two consecutive maximal conductance values (adjacent family members); thus, for each family a curve was plotted. We developed Matlab scripts to automatically analyze each such family curve. First, our scripts checked whether a family curve was monotonic or not, by simply comparing the burst characteristic values of each two adjacent family members (i.e., by calculating their difference). If, for a family, all these differences have the same sign (either positive or negative), the family curve is considered monotonic; otherwise, it is considered nonmonotonic. We separated the families into subgroups according to their monotony (e.g., all four member h families showing monotonically decreasing curves corresponding to period were grouped together). Then, our scripts calculated the average change in burst characteristic for each such subgroup of families (sum over all families of the difference of the last and first members’ burst characteristic values in each family divided by the number of families).

#### Increasing ḡ_h_ increases the spike frequency and decreases the period

**Figure 7.**
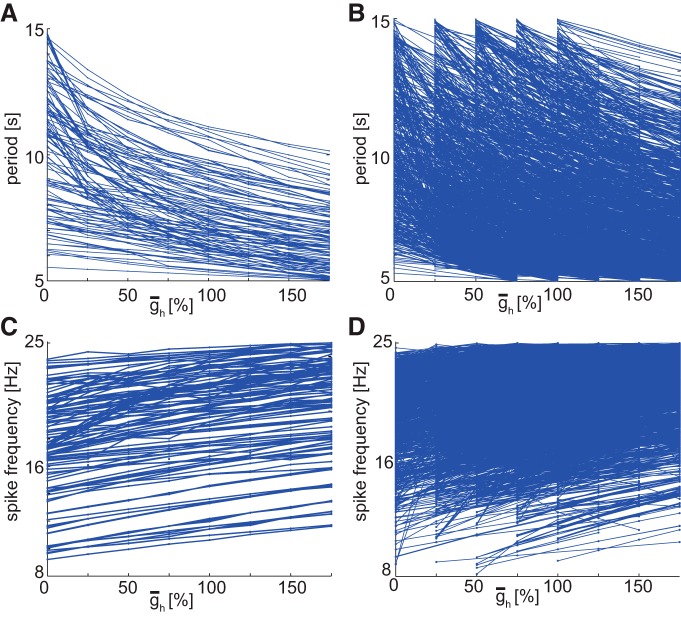
Variations of burst characteristics (period and spike frequency) vs ¯g_h_ for the realistic HCOs. Lines connect adjacent family members. ***A***, Period vs ¯g_h_ for h families with eight members (144 families). All curves are monotonically decreasing. ***B***, Period vs ¯g_h_ for h families with four members (4,322 families). For four-member families, regardless of the beginning point, all curves are monotonically decreasing. ***C***, Spike frequency vs ¯g_h_ for h families with eight members. Most of the curves are monotonically increasing. ***D***, Spike frequency vs ¯g_h_ for h families with four members. For four-member families, regardless of the beginning point, the vast majority of curves are monotonically increasing.

Figure 7, *A* and *B*, shows the period plots obtained for h families with eight members and with four members, respectively, for the *r*HCO group. However, we obtained similar plots for all possible h family sizes of the *r*HCOs. For all families of h, such plots (Fig. 7*A*,*B*) showed that increasing the maximal conductance (ḡ_h_) of the hyperpolarization-activated current monotonically decreases the burst period of the *r*HCOs, which confirms and extends the results in the study by Hill et al. (2001) stated for the canonical model when one parameter was varied at a time (more restricted parametric space). This decrease occurs because of the ability of the h current to depolarize the inhibited neuron and advance the transition to the burst phase.

Figure 7, *C* and *D*, shows the spike–frequency plots obtained for h families with eight members and with four members, respectively, for the *r*HCO group. For most families of h (except for 8 out of 2,703 families with five members, for 22 of 4,322 families with four members, for 63 of 6,325 families with three members, and for 353 of 9,895 families with two members), spike–frequency plots (Fig. 7*C*,*D*) revealed that increasing the maximal conductance (ḡ_h_) of the 560 hyperpolarization-activated current monotonically increased the spike frequency of the *r*HCOs. As the family size increased, the average increase in spike frequency for each family increased, from ∼0.29 Hz for h families with two members to 0.71 Hz for h families with eight members (amount calculated as the ratio between the average increase in frequency for all families having same number of members and the number of families). However, as stated in the previous section, increasing ḡ_h_ did not increase the burst period but decreased it, due to the ability of *I*_h_ to promote the escape of the inhibited neuron. The increase in spike frequency happens because by increasing ḡ_h_ the inhibited neuron escapes earlier and starts bursting earlier, which makes the burst duration shorter, leading to higher average spike frequencies.

#### Increasing ḡ_CaS_ increases both period and spike frequency

For most families of CaS, such period plots (data not shown) revealed that increasing the maximal conductance of the slow Ca current (ḡ_CaS_) monotonically increased the cycle period of the *r*HCOs. For many of these families, the increase was almost linear. However, for few CaS families—2 families (of 929) of four members, 35 families (of 5,263) of three members, and 137 families (of 17,851) of two members—increasing ḡ_CaS_ decreased (not monotonically) the period. We conclude that these results confirm and extend the previous results in the study by Hill et al. (2001).

For all families of CaS, spike–frequency plots (data not shown) revealed that increasing the maximal conductance of the slow Ca current (ḡ_CaS_) monotonically increased the spike frequency of the *r*HCOs. For most families, the increase was big (with an average increase from 5 Hz for families with three members up to >11 Hz for families with six members). This result confirmed the results in the study by Hill et al. (2001) regarding the influence of CaS on spiking activity of the canonical model and explains the effect on period because increased spike frequency leads to greater inhibition of the opposite cell of the HCO.

#### Increasing ḡ_P_ increases both period and spike frequency

For most (except for 34 of 3,048) families (of two members) of P, plots (data not shown) revealed that increasing the maximal conductance of the persistent Na^+^ current (ḡ_P_) increased the period of the *r*HCOs. For many of these families, the increase was large (>9.77 s); the average (over all families with two members) increase of the period was 4.255 s.

For the *r*HCOs, spike–frequency plots (data not shown) of P families revealed that increasing the maximal conductance of the persistent Na^+^ current (ḡ_P_) increased the spike frequency. For many P families, the increase was noteworthy, with an average increase of 5.37 Hz. The effect on spike frequency accounts for the effect on period through increased inhibition of the opposite cell of the HCO.

#### Increasing ḡ_K2_ decreases the period

For the *r*HCOs, plots (data not shown) of period versus ḡ_K2_ values for all families of K2 revealed that for most (except for 19 of 13,163 families with two members) families of K2, increasing the maximal conductance of the persistent K current (ḡ_K2_) monotonically decreased the period of the *r*HCOs. For many of these families, the decrease was large (average of 3.32 s for families with two members, of 4.87 s for families with three members, and of 6.59 s for families with four members). Once again, our results confirm and extend the previous results of the study by Hill et al. (2001).

For many families of K2 (except for 424 of 13,163 families with two members, and for 4 of 1,299 families with three members), spike–frequency plots (data not shown) showed that an increase in the maximal conductance of the persistent K current (ḡ_K2_) led to a decrease in the spike frequency. The decrease was >2 Hz on average, and it occurs due to the ability of *I*_K2_ to decrease the peak of the (slow-wave) oscillation during the burst. Again, the change in spike frequency with ḡ_K2_ accounts for the change in period due to a change in inhibition of the opposite cell in the HCO.

#### Increasing ḡ_Leak_ decreases both period and spike frequency

For the *r*HCOs, plots (data not shown) of period versus ḡ_Leak_ values for all Leak families revealed that for most (except for 345 of 11,873 families with two members, for 1 of 2,133 families with three members, and for 1 of 452 families with four members) families increasing ḡ_Leak_ decreased the period. The average decrease of the period was 2.46 s for families with two members and went up to 5.78 s for families with six members.

For most Leak families (except for 13 of 11,873 families with two members), spike–frequency plots (data not shown) of spike frequency versus ḡ_Leak_ values revealed that increasing ḡ_Leak_ decreased the spike frequency. The average decrease of the spike frequency was 2.72 Hz for families with two members, and it increased up to 7.55 Hz for families with six members. Again, the change in spike frequency with ḡ_Leak_ accounts for the change in period due to a change in inhibition of the opposite cell in the HCO.

#### Increasing ḡ_SynG_ results in negligible changes in period and spike frequency

Plots (data not shown) of period versus ḡ_SynG_ values for all *r*HCO families of SynG revealed that for many families (except for 1,241 of 7,047 families with two members, for 1,099 of 5,086 families with three members, for 1,114 of 4,124 families with four members, for 1,038 of 3,046 families with five members, for 799 of 1,971 families with six members, for 572 of 1,061 families with seven members, and for 761 of 1,023 families with eight members) increasing ḡ_SynG_ increased the period. As the number of members within families increased, there was an increase in the number of families for which increasing ḡ_SynG_ decreased the period. However, the change (either increase or decrease) in period was small (<0.4 s on average), and therefore we conclude that SynG does not play an important role in controlling period.

For most families of SynG, spike–frequency plots (data not shown) revealed that increasing ḡ_SynG_ changed the spike frequency of the *r*HCOs negligibly (average change between 0.18 and 0.31 Hz for families with two and seven members, respectively). For most families (except for 2,291 of 7,047 families with two members, for 1,522 of 5,086 families with three members, for 999 of 4,124 families with four members, for 704 of 3,046 families with five members, for 409 of 1,971 families with six members, for 214 of 1,061 families with seven members, and for 170 of 1,023 families with eight members), increasing ḡ_SynG_ slightly increased the spike frequency. We conclude from the small changes in burst characteristics (period and spike frequency) when ḡ_SynG_ is varied that, while some amount of graded transmission may contribute to alternating bursting (maybe 25% is good enough), there is no added benefit to having higher ḡ_SynG_ values.

#### Increasing ḡ_SynS_ increases the period, but decreases the spike frequency

For almost all *r*HCO families of SynS, plots (data not shown) revealed that increasing the maximal conductance of the spike-mediated synapse (ḡ_SynS_) monotonically increased the burst period of the *r*HCOs (except for 74 of 16,950 families with two members, and for 3 of 6,390 families with three members). The average increase of period was from 2 s for families with two members to 5 s for families with six members.

For many SynS families (>55%), spike–frequency plots (data not shown) showed that increasing the maximal conductance of the spike-mediated synapse (ḡ_SynS_) decreased the spike frequency of the *r*HCOs. The decrease started from an average of 0.35 Hz for families with two members, and it increased to an average of 2.5 Hz for families with six members. The larger the family size, the larger was the number of the families that show this decrease in spike frequency (from 55% of families with two members to 90% of families with six members).

### Analysis of family structure shows how ḡ_h_ influences period of realistic HCO instances

To analyze how burst characteristics in *r*HCOs are influenced by varying a ḡ value, we took advantage of family structure and made graphs like those in Figure 7, in which we plotted a burst characteristic [e.g., period (Fig. 7*A*,*B*) or spike frequency (Fig. 7*C*,*D*)] vs ḡ values for families of different sizes. By looking across families, we could spot trends in these relationships. We will focus here on period versus ḡ_h_ values for h families of *r*HCO instances. All these plots show monotonically decreasing curves of period versus ḡ_h_ values for each h family, regardless of family size (we checked the monotonies using our Matlab scripts). We observed that these monotonically decreasing curves show different rates of decline, indicating different sensitivities of period to changes in ḡ_h_. Some seemed to decline very steeply with increasing ḡ_h_, and some seemed to decline quite gradually.

**Figure 8.**
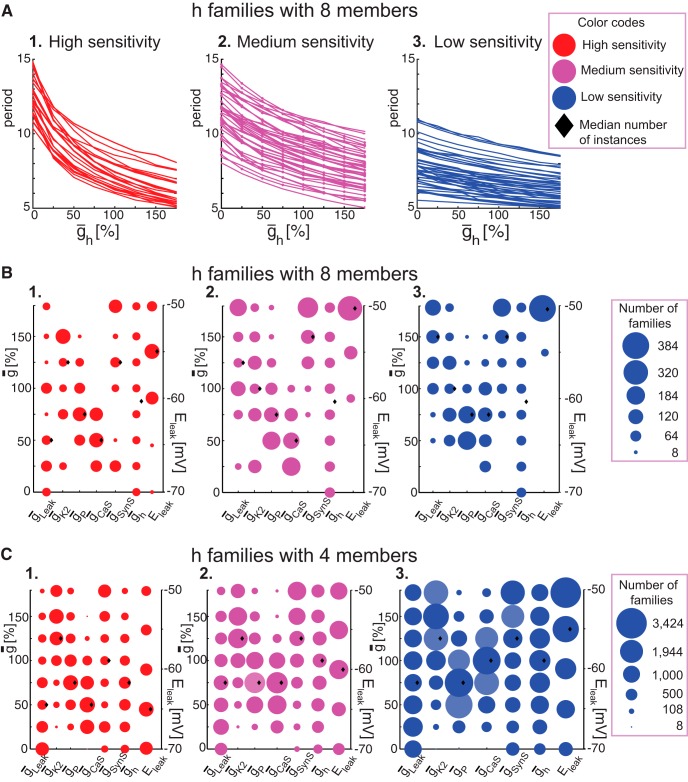
Sensitivity of period to the variation of ¯g_h_ for h families of realistic HCOs. ***A***, Plots of period vs ¯g_h_ for h families with eight members (144 families). Lines connect adjacent members of an h family forming a curve. Curves were split into three groups according to their period sensitivity (slopes), as follows: high (steep slopes; 1); medium (medium slopes; 2); and low (shallow slopes; 3). ***B***, Parameter values vs the number of distinct families for all h families with eight members separated into these three groups of sensitivity. The size of each point quantifies how many different families have that parameter value (here, we scale up the sizes by five times for a better display). Black diamond shapes show the median values for each parameter. ***C***, Parameter values vs the number of distinct families from all h families with four members separated into the three groups of sensitivity. For simplicity, plots do not show data for ¯g_SynG_ since the graded transmission did not have a significant influence on burst characteristics (see Sensitivity of period to variations of ¯g's, and Sensitivity of spike frequency to variations of ¯g's).

Using our own Matlab scripts (see Sensitivity classification), we automatically separated the h families according to their curve steepness (period sensitivity) into the following three groups: high-sensitivity (steep slope), medium-sensitivity, and low-sensitivity (shallow slope). Figure 8*A* shows the split between the curves of the h families with eight members whose curves were illustrated in Figure 7*A*. Our algorithm found 35 families with high-sensitivity, 57 with medium-sensitivity, and 52 with low-sensitivity. Table 3 provides the number of curves for each sensitivity group for all h families of *r*HCOs. It is easy to see that the group with steep slopes had the smallest number of families (curves). Most families with many (six to eight) members were in the medium-sensitivity group, otherwise the low-sensitivity group prevailed. For each sensitivity group (high, medium, and low), the plots in Figure 8*B* show the ranges of each parameter for h families with eight members. Plots in Figure 8*C* show the parameter ranges for the h families with four members (Fig. 7*B*, family curves). In the plots of Figure 8, *B* and *C*, the size of each point shows how many distinct families of the respective family group have a certain parameter value. For better visualization, the points in Figure 8*B* have been scaled up five times because there was a small number of families for many parameter values, and black diamond shapes show the median values for each parameter.

**Table 3:** The number of h family curves of the *r*HCOs classified according to their (period) sensitivity (curve slopes)

	Three members	Four members	Five members	Six members	Seven members	Eight members
High	690	684	583	323	121	35
Medium	1,002	1,239	979	528	233	57
Low	4,633	2,399	1,141	434	183	52

For the high-sensitivity group (Fig. 8 *B1*,*C1*), the reversal potential E_leak_ showed a full range. For h families with many members (six to eight members), E_leak_ (−55 or −60 mV) showed the greatest number of families; for the rest of h families (with three to five members), a hyperpolarized E_leak_ (−65 or −70 mV) showed a slight increase in the number of families. ḡ_Leak_ showed also a full range of its values, with an increase in the number of families as ḡ_Leak_ decreases. ḡ_K2_ had values between 25% and 175%, which meant that K2 must be present for *r*HCO h families to have high h sensitivity. An increased amount of ḡ_K2_ seemed to increase the number of families, with a maximum reached at 150% (median, 125%). ḡ_P_ had a range similar to ḡ_K2_, with values between 25% and 175% (median, 75%). ḡ_CaS_ showed a full range of its values for h families with three members, and a range between 25% and 150% for the other h families. More interestingly, for ḡ_CaS_ values between 25% and 75%, we observed the most h families, while for higher values we observed just a few families (peak at 50%, which was also the median value). ḡ_SynS_ seemed to be needed (at least 25% present) to have full stability (eight member families); otherwise, it showed the whole range of values (e.g., families with four members; Fig. 8*C1*). ḡ_SynG_ and ḡ_h_ showed the full range, with an almost equal number of families at each value.

For the medium-sensitivity group (Fig. 8*B2*,*C2*), the reversal potential E_leak_ showed a full range for all h families except the ones with eight members, for which the range was from −60 to −50 mV. There was a monotonic decrease in the number of families as the reversal potential becomes hyperpolarized. ḡ_Leak_ showed a full range for its values for all h families except those families with eight members, for which the range was from 25% to 175%. ḡ_K2_ had values between 25% and 175%, except for one case of a family with five members, which meant that K2 must be present to have *r*HCOs h families with medium-sensitivity. An increased amount of ḡ_K2_ seemed to increase the number of families, with a maximum reached at 150%; the median value was at 125%. ḡ_P_ had a range similar to that of ḡ_K2_, with values between 25% and 175% (median, 75%). ḡ_CaS_ showed a range between 25% and 175% for the h families with three to seven members, and a range between 25% and 100% for the families with eight members. ḡ_SynS_ seemed to be needed (at least 75% present) to have full stability (eight-member families), otherwise it showed a whole range of values. ḡ_SynG_ and ḡ_h_ showed the whole range of values for all families of different sizes.

For the low-sensitivity group (Fig. 8*B3*,*C3*), the reversal potential E_leak_ showed a full range for families with fewer than seven members, and a range between −60 and −50 mV for families with many members (seven and eight members). More interestingly, for this sensitivity group there was a monotonic decrease in the number of families as the reversal potential becomes hyperpolarized. ḡ_Leak_ showed a full range for families with fewer than seven members. ḡ_K2_ had values between 25% and 175% for h families with six to eight members, and a full range for the remaining h families (three to five members), although analysis showed a very small number of families where there is no K2 (Fig 8*C3*, this point in the plot is too small to see and represents one family). An increased amount of ḡ_K2_ seemed to increase the number of families, and the median value was at 125%. ḡ_P_ had values between 25% and 175% for families with five to eight members, and full range for the remaining h families; the median value was 75%. ḡ_CaS_ showed a full range for families with three members, and a range between 25% and 175% for the remaining h families. The median values were at 100% for families with three to five members and at 75% for the others (six to eight members). ḡ_SynS_ seemed to be needed (at least 75% present) to have full stability (eight-member families), otherwise it showed the whole range of values (data not shown). ḡ_SynG_ and ḡ_h_ showed the whole range for all families of different sizes.

There is no set or range of parameters that we can detect that characterizes the period sensitivity (period vs ḡ_h_). Moreover, we observed no linear correlations of parameters in any of the period sensitivity groups. For example, we applied principal component analysis (PCA) to each of the three sensitivity subgroups of eight-member h families of *r*HCOs in Figure 8, *A* and *B* (data not shown), and found no linear correlations.

There are, however, a few observations that we can make from the plots in Figure 8 about how parameters contribute to period sensitivity. The graded synapse ḡ_SynG_ seems to not have any influence on period sensitivity as varying it does not change the number of families (constant). ḡ_K2_, ḡ_P_, and ḡ_CaS_ seem to be needed to produce *r*HCOs (values of at least 25%). As expected, E_leak_ and ḡ_Leak_ seem to affect activity in the same way; the number of *r*HCOs increases for high sensitivity or decreases for low sensitivity for both parameters at the same time. ḡ_K2_ and ḡ_P_ also affect activity but differently, as it seems that one compensates for the effect of the other; this result (correlated triplet of ḡ_Leak_, ḡ_K2_, and ḡ_P_) was observed (Doloc-Mihu and Calabrese, 2014) in regular bursting neurons (isolated HCO cells). The amount of ḡ_CaS_ does seem to have an effect on sensitivity type. There are few high-sensitivity h families with three members with zero ḡ_CaS_ and few low-sensitivity h families with three members with zero ḡ_K2_ and ḡ_P_. Except for h families with three members where it shows the full range from 0% to 175%, it seems that the amount of ḡ_CaS_ shows the smallest range for the high-sensitivity group (25–75%) for families with eight members, and 25–150% for families with four to six members, with most families having values between 50% and 100%. Except for families with eight members, the low- and medium-sensitivity families show a range of 25–175% for ḡ_CaS_. For the high-sensitivity families with different numbers of members, the median is consistently at 50% of ḡ_CaS_; for medium-sensitivity families, it is at 50% for h families with many members (seven and eight), and at 75% for the remaining h families (3-6 members); and for the low-sensitivity families, it is either at 75% or at 100% of ḡ_CaS_.

### Analysis of family structure helps identify ensembles of model instances for modeling studies

Our family analysis can help to identify parameter sets that approximate the physiological activity of any neuronal or network model more closely than hand-tuning methods and more efficiently than search methods, such as genetic algorithms (Holland, 1992). We propose selecting these robust parameter sets from the noninterrupted families with a large number of members (more than four members); each family identifies one such potential set of parameters. Our first step for identifying a good ensemble of model instances to best approximate the HCO physiological bursting was to select from the set of noninterrupted large member families a subset that have nonzero values for each parameter (i.e., ḡ ≠ 0) other than the defining parameter. For example, there were 119 such families from the 144 h families of realistic HCOs with eight members. Then, for our next step we selected from the above subset only those families that include members whose corresponding isolated neurons show a specific activity type.

**Figure 9.**
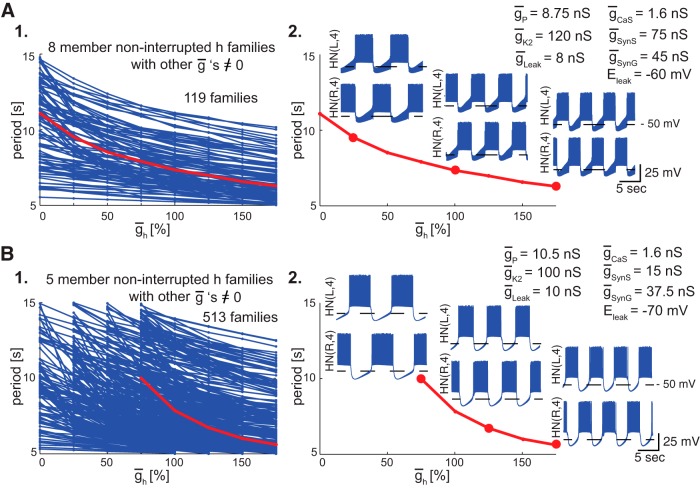
Examples of ensembles of physiological model instances with large noninterrupted families from which one can choose a canonical model to reflect typical robust physiological activity. Plots show period vs ¯g_h_ values for h families of realistic HCOs with lines connecting adjacent family members. The chosen ensemble is highlighted in red. The voltage traces of the two interneurons corresponding to a certain model from the family are shown above their corresponding ¯g_h_ values (bigger red circles); each trace is the same amount of time (22 s). Horizontal lines indicate −50 mV for each trace. ***A1***, Ensemble corresponding to a noninterrupted h family with eight members (of 119 families having all other ¯g’s ≠ 0) showing medium-sensitivity slope curve of period vs ¯g_h_. ***A2***, The three selected models give rise to spiking isolated neurons by cutting any synaptic transmission (¯g_SynS_ = 0 and ¯g_SynG_ = 0). ***B1***, Ensemble corresponding to a noninterrupted h family with five members (out of 513 noninterrupted families having all other ¯g values ≠0) showing a high-sensitivity slope curve of period vs ¯g_h_. ***B2***, The three selected models give rise to realistic bursting isolated neurons by cutting any synaptic transmission between the two neurons.

For example, in Figure 9, *A1* and *B1*, we show (in red) two h families of realistic HCOs selected from the families with eight and five members, respectively. We selected these families such that the isolated neurons corresponding to their members have spiking and realistic bursting, respectively. In Figure 9, *A2* and *B2*, we show the voltage traces of both neurons for some members of these two h families; 22 s was represented in each trace. One can see how varying ḡ_h_ influences the bursting activity. The first family (Fig. 9*A*) shows a moderate sensitivity to variations of ḡ_h_, and the second one (Fig. 9*B*) shows a high sensitivity (busting speeds up fast by modulating h).

The voltage traces shown in Figure 9, *A2* and *B2*, illustrate activity closely corresponding to leech physiological HCO activity (period between 5 and 15 s; average spike frequency between 8 and 25 Hz; duty cycle between 50% and 70%), as indeed each member of a *r*HCO family must. Model instances from the family in Figure 9*A2* are of particular interest to us since they have the strong synaptic inhibition, observed experimentally. By having a duty cycle <50% (49.38%), the canonical model of Hill et al. (2001) would not be included in our realistic HCO group but in the functional HCO group (it belongs to the set of h families of *f*HCOs with six members). Like the canonical model of Hill et al. (2001), our model instances from Figure 9*A2* have isolated neurons with spiking activity type.

All the h families of *r*HCO model instances from Figure 9*A1* thus better approximate the physiological activity of the leech HCO system than the canonical model of Hill et al. (2001), because every member of every family conforms to the physiological activity. Similarly, other large h families of *r*HCOs (Fig. 9*B1*, four-member families) conform to the physiological activity. These families thus comprise a useful ensemble of model instances for exploring the mechanisms of the physiological activity. We conclude that our family-based method is a good tool for identifying such ensembles.

## Discussion

We used an HCO model that replicates the rhythmic alternating bursting of mutually inhibitory interneurons of the leech heartbeat CPG to investigate the robustness (or sensitivity) and modulability of bursting activity to maximal conductance (ḡ) variations. We systematically explored the parameter space of two groups from the 10.5 million instances comprising the entire HCO model space, whose characteristics were previously recorded into a database, as follows: *r*HCOs with 99,066 instances; and *f*HCOs with 1,103,073 instances (Doloc-Mihu and Calabrese, 2011). To analyze such a large number of instances, we developed a new method (family based) that allowed us to classify instances from these two large groups (*r*HCOs and *f*HCOs) into much smaller subgroups of instances called families. Families are groups of model instances that differ only by the value of the one parameter that defines the family. By examining family structures and patterns, we developed new measures of robustness (or sensitivity) of bursting activity to changes in model parameters and investigated how such variations can be harnessed to modulate activity characteristics without compromising robustness. These measures can be easily adapted to other forms of electrical activity and other parameter types, and applied to brute-force databases. Our decision to limit our parameter variations to variations of ḡ values while holding constant other kinetic parameters of the ionic currents was based on our available voltage-clamp analyses, which used average data to determine activation/inactivation and temporal characteristics of the currents. This decision limits the impact of our analysis (Amendola et al., 2012) but not the ability to apply our method to other systems where varying these parameters seems appropriate.

### Robustness measures

We investigated independently the role of each conductance in the robust maintenance of functional bursting activity. Families organize model instances (*r*HCOs and *f*HCOs) into small subsets of instances sharing the same seven parameter values. Using families, we defined robustness with a number of different measures and analyzed all families of each parameter with respect to these measures to find potential patterns.

Our first measure was based on the family size: the greater number of these large families, the greater robustness. *r*HCO families with many members (seven or eight members of possible eight members) all maintain realistic bursting activity over a large range of values of the parameter. By this measure, realistic HCO activity was robust to changes in ḡ_h_ and ḡ_SynG_, but it was very sensitive to changes in ḡ_P_, and to a lesser extent to changes in ḡ_K2_. Our previous results (Doloc-Mihu and Calabrese, 2014) show that isolated neurons that are realistic bursters had only CaS families with one to three members, meaning that the activity of the realistic bursters was quite sensitive to changes in the value of ḡ_CaS_. By adding inhibition (HCO configuration), the number of CaS families with many members (one to six) increased, making the system less sensitive to variations in the value of ḡ_CaS_. However, the small number (four) of CaS families with six members for *r*HCOs suggested that the system is not as robust to variations in ḡ_CaS_ values as it is to variations in ḡ_h_ values.

Our second measure of robustness was based on family sequence (the sequence of values of the varied parameter within the family), as follows: a noninterrupted family sequence shows robustness (not broken by changing the defining family parameter value over its permissible range) and an interrupted family sequence shows sensitivity (susceptible to a change in activity type or nonphysiological burst characteristics by changing the defining family parameter values). We found that the higher the values of ḡ_h_ within the family, the more robust the sequence; thus, the higher likelihood of finding realistic HCO activity. Conversely, the lower the values of ḡ_h_ within the family, the more sensitive the sequence. Lower ḡ_Leak_ values (0–100%) resulted in more robust realistic HCO activity (more noninterrupted families than for higher ḡ_Leak_ values). Stronger (≥60 nS) spike-mediated synaptic transmission promoted robust realistic HCO activity. The system is quite robust to variations of graded transmission (SynG).

Our third measure of robustness was based on the activity type shown by the missing family members in both interrupted and noninterrupted families (i.e., both within and outside the permissible range): best if missing members have functional HCO activity, because by varying the parameter the HCO bursting is still kept functional. Plots of distributions of missing points from h families of *r*HCOs revealed that HCO bursting activity was robust to variations of ḡ_h_, as variations of ḡ_h_ maintained *f*HCO bursting activity, though they might interrupt or terminate a realistic HCO family sequence. We conclude that ḡ_h_ is a potential target for modulation, since the HCO system shows robustness to its variations. The h families of the realistic bursters group (isolated neurons) were in general small (64 families with two and three members) with very few larger families (6 families with four members and 1 family with five members), with most missing members silent (data not shown; Doloc-Mihu and Calabrese, 2014). A change in ḡ_h_ has a significant chance of disrupting regular bursting activity in synaptically isolated neurons transforming their activity into silence, but by adding inhibition and forming an HCO, such variations of ḡ_h_ maintain functional HCO bursting activity. Based on these observations, it seems that in the heartbeat HCO inhibition makes the activity very robust to variations of ḡ_h_.

Plots of distributions of missing points from CaS families of *r*HCOs revealed that HCO bursting activity was robust to variations of ḡ_CaS_, if ḡ_CaS_ was ≥75% of the canonical value. Realistic HCO activity is robust with a low ḡ_Leak_ (0–100%) present in the system (missing members have mostly *f*HCO activity); ḡ_Leak_ >100% favors spiking and silent activity. The distribution of missing points from P families of *r*HCOs revealed that changes in the amount of ḡ_P_ moved the HCO bursting activity outside the *f*HCO bursting range (to spiking, silent, or asymmetric activity). We conclude that ḡ_P_ is not good as a target for modulation since the activity type of the system is very sensitive to its variations. Changes in the amount of ḡ_K2_ also disrupted HCO bursting activity, but not quite as severely as ḡ_P_. Finally, distributions of the missing members from SynS families for the *r*HCOs show that at least 100% of ḡ_SynS_ was necessary to maintain *f*HCO bursting. Strong spike-mediated synaptic transmission promotes functional HCO activity.

For h families of *r*HCOs, most of the missing members (increasing percentage, from 62.3% for families with two members to 85.6% for families with seven members) have *f*HCO bursting activity for which only their duty cycle is outside the permissible physiological range (0.5–0.7). This result pertains to all h families of *r*HCOs (Table 3). The percentage of these missing members of h families that had burst periods either too long or too short to have *r*HCO activity (only their period outside the permissible range of 5–15 s) varied between 6.8% for families with seven members and 10.3% for families with five members. The number of missing members that spike too slowly (spike frequency <8 Hz) or too quickly (spike frequency >25 Hz) during the burst diminishes (from 8.7% for families with two members to 2% for families with seven members). For all h families, <0.3% of missing members from h families of *r*HCOs went outside the permissible realistic HCO range for all three criteria.

A corollary of our measures of robustness, which directly indicates the suitability of a parameter for functional modulation of the bursting activity, was based on a reliable and predictable (monotonic) variation in burst characteristics (period and spike frequency) within the realistic and functional range when a parameter was individually varied. Several clear patterns have emerged for *r*HCOs. Increasing ḡ_h_ increased the spike frequency moderately and decreased the period, speeding up the realistic HCO bursting activity. Increasing ḡ_CaS_ increased the spike frequency more strongly and increased the period. Increasing ḡ_P_ strongly increased the spike frequency and the period. For many K2 families, increasing ḡ_K2_ decreased the spike frequency and the period. For most Leak families, increasing ḡ_Leak_ decreased the spike frequency and the period. Increasing ḡ_SynG_ showed negligible changes in spike frequency and period, and increasing ḡ_SynS_ decreased the spike frequency and increased the period. All these results on the influence of parameter variation on burst characteristics confirmed and extended the previous results in the study by Hill et al. (2001) obtained in a more restricted parametric space (variation of a single parameter over a range similar to the one presented here but on a background of canonical values for all other parameters). So, to decrease period, for example, we can increase the amount of ḡ_h_, ḡ_K2_, and ḡ_Leak_ or decrease the amount of ḡ_CaS_, ḡ_P_, and ḡ_SynS_. If we consider the other measures of robustness and this measure of modulatory effectiveness, then varying ḡ_h_ appears ideal for modulating period because the HCO system is very robust to the variation in ḡ_h_ and ḡ_h_ consistently and substantially modulated period with minimal adverse effects on spike frequency or duty cycle.

For a specific (chosen) parameter, t, we propose the following simple formula as a general measure of robustness for large databases:

(1) $$R_{t,n}=w_{X}*X_{t,n}+w_{Y}*Y_{t,n}+w_{Z}*Z_{t,n}$$

where $X_{t,n}$ is the number of families with more than a chosen number of members (n, best when n is large); $Y_{t,n}$ is the number of noninterrupted families with more than n members; $Z_{t,n}$ is the number of interruptions (missing families members) that do not change the activity type; and $w_{X},\;w_{Y},\;w_{Z}$ are chosen weights that show the importance of each individual robustness measure in assessing the final robustness of the parameter t, with $w_{X}+\;w_{Y}+\;w_{Z}=1$. The formula is a simple weighted cumulative sum of the three measures of robustness that we proposed above. The weights indicate the importance of each measure of robustness within the system and depend on what the user values as robust in their system.

In our study here, we chose to assess the robustness for the realistic HCOs. We selected as a parameter t, the maximal conductance of the hyperpolarization-activated cation (h) current, ḡ_h_. For n=4, our numbers for calculating total robustness follow. First, we selected from our *r*HCOs all h families with more than four members and obtained the first measure of robustness, $X_{h,4}\;=4,669$. The total number of noninterrupted families is $Y_{h}\;=11,210$, and the number of interruptions that keep the functional bursting activity, *f*HCO is $Z_{h}=\;98,882.$ Next, $Y_{h,4}\;=1,729$, and $Z_{h,4}\;=8,945$. Finally, we chose to assess robustness by giving equal importance to our first two measures and by not considering the third one (i.e., $w_{X}=\;w_{Y}\;=0.5,\;{\;w}_{Z}=0$, and $R_{h,4}=3,199$). To put this number in perspective, it can be normalized to the user’s goal. For example, normalizing to the total number of families with more than four members shows that ∼70% (0.685) of the h families with more than four members maintain realistic alternating bursting activity when ḡ_h_ varies (i.e., no interruptions).

### Robustness and parameter correlations

We emphasize that we previously found no linear correlations among parameters in the *r*HCO group (Doloc-Mihu and Calabrese, 2014). Figure 1 reveals a nonlinear correlation among E_leak_, ḡ_Leak_, ḡ_K2_, and ḡ_P_ for the *r*HCOs (c.f. Goldman et al., 2001), but no other nonlinear correlations were observed. Nor were there any linear correlations (via the PCA method) or other nonlinear correlations in any subgroup tested. For example, we applied PCA to each of the three sensitivity subgroups of eight-member h families of *r*HCOs of Figure 8, *A* and *B* (data not shown), and found no linear correlations.

How then would parameter correlations affect robustness as determined by our cumulative measure? Our previous work showed that for the component neurons of the HCO to be endogenous bursters, there must be a strict linear correlation among ḡ_Leak_, ḡ_K2_, and ḡ_P_. We enforced this correlation in our database by requiring endogenous bursting of the component neurons in all families considered. We then used the same measures as above and obtained $X_{h,4}^{enf}=53,\;Y_{h,4}^{enf}=51$, $Z_{h,4}^{enf}=2$, and $R_{h,4}^{enf}=52$. These numbers indicate that this correlation, should it be biologically enforced, would limit the robustness. This finding is consistent with the observation that endogenous bursting in heart interneurons is very sensitive to changes in leak (e.g., as caused by sharp microelectrode penetration) and that it is not necessary for robust alternating bursting activity (Sorensen et al., 2004) or when h current is modulated (Tobin and Calabrese, 2006). Moreover, the vast majority [94,487 (95.37%)] of *r*HCOs in our database are made up of component neurons that are spiking. It is interesting to note that such biologically enforced correlations have been observed in the stomatogastric nervous system (Goaillard et al., 2009; Tobin et al., 2009) and cardiac ganglion of crustaceans (Ball et al., 2010). In the cardiac ganglion at least, such correlations appear to increase robustness.

### Period sensitivity

We separated our h families with eight members into three groups according to their period sensitivity to increasing ḡ_h_ (high, medium, and low sensitivity). For high-sensitivity families, increasing ḡ_h_ speeds up bursting strongly; a large decrease of period with increasing ḡ_h_ occurs (typically before 50% ḡ_h_), then this decrease moderates at higher ḡ_h_ values. For low-sensitivity families, increasing ḡ_h_ speeds up bursting more uniformly; period decreases moderately but almost linearly for all ḡ_h_ values. The medium-sensitivity families are intermediate; period decreases steadily with increasing ḡ_h_, but there is neither a sudden drop nor a range of weak period decrease. This splitting seems functional to us and shows how parameters interact within specific ranges to produce these different types of sensitivities.

We put in the medium-sensitivity group all those families that were in neither the high-sensitivity nor the low-sensitivity group. One could argue that defining this group of medium sensitivity might not be germane to the analysis, because the set of h families illustrates period curves whose slopes (with respect to the horizontal axis) occupy the entire spectrum of angles. The decision to keep three groups versus two groups (high and low-sensitivity) was based on visual inspection of 100 randomly selected slopes. This process helped us to set the criteria for slope angles of the two important cases of low and high-sensitivity. The results show that the region with the steepest slope (high-sensitivity region) can appear anywhere, but in ∼98% of the cases it appears at the very beginning of the curve [i.e. at low values of ḡ_h_ (<100%)]. For the medium-sensitivity group, a steep slope region occurs at higher ḡ_h_ (100–150%) or not at all. For the low-sensitivity group, there is no steep slope region, only a nearly constant slope. Note that all curves show monotonically decreasing periods with the increase in ḡ_h_.

Splitting the families into three sensitivity groups, moreover, helped us to define how other parameters define period sensitivity to the variation of ḡ_h_ by analysis of the ends of a spectrum. We used this analysis of h families to investigate the effects of background conductances on the period of realistic HCO bursting. To sum up this period sensitivity analysis, several parameters influence the realistic HCO burst period. Shorter periods seem to be influenced by K2 and P working together against Leak, with CaS also having some effect. Longer periods seem to be affected by K2 and E_leak_ working together against P, by the amount of CaS, which has the same influence as K2, and also by the spike-mediated synaptic transmission SynS. From this analysis, we conclude that period is influenced by groups of parameters but it is easily and predictably controllable by modulating ḡ_h_.

It appears that in the HCO system there are several different mechanisms that influence robust realistic bursting. One mechanism that we found involves E_leak_, *I*_Leak_, *I*_P_, and *I*_K2_ working together to compensate for the variations in each to keep bursting functional. In isolated bursting neurons, these parameters interact in a linearly correlated way (Doloc-Mihu and Calabrese, 2014). Linking these isolated bursting neurons by mutually inhibitory synapses into HCOs increases the system robustness and causes these parameters to interact in a nonlinear way.

Another mechanism we found involves specific parameters working individually to change the burst characteristics (here, we focused on burst period and spike frequency). Our results show that realistic, physiological bursting activity is robust to changes in the amount of h current, and that h is a great target for modulating period (because varying the amount of ḡ_h_ changes the burst period in a significant and predictable way). This result confirms and supplements the conclusions of previous theoretical studies (Nadim et al. 1995; Hill et al., 2001) and extends them to combinations of key parameters that are varied together. Moreover, the peptide myomodulin upmodulates the h current in heart interneurons that comprise HCOs and consistently speeds the burst period (Masino and Calabrese, 2002; Tobin and Calabrese, 2006). Recent experiments (Tsuno et al., 2013) show that *I*_h_ is a good modulation target for the phase of neurons in rat cortex, emphasizing the general importance of this current for modulation.

### Applicability of the robustness measures

Last, we emphasize that the measures of robustness we developed here are easily adaptable to other neuronal and network models. Brute-force databases, such as HCO-db, lend themselves to the family analysis we performed here. By identifying families and applying our measures—the number of large families, the number of noninterrupted families, and the missing family members that show functional albeit not physiological activity (i.e., no change in activity type)—one can identify parameters that can be safely modulated. Then, using large families one can identify those parameters that consistently modulate an adaptable and desirable activity characteristic. Family analysis can also allow the identification of robust parameter sets that more closely approximate physiological activity of any neuronal or network model, and thus allow the construction of an ensemble of physiological model instances for further mechanistic studies. For example, the ensemble of physiological model instances with large noninterrupted families we have identified will allow us to ask whether for effective, functional modulation there are advantages to covarying currents over a single particularly efficacious current like h in the leech heartbeat HCO.

## Synthesis

The decision was a result of the Reviewing Editor Marlene Bartos and the peer reviewers coming together and discussing their recommendations until a consensus was reached. A fact-based synthesis statement explaining their decision and outlining what is needed to prepare a revision is listed below. The following reviewers agreed to reveal their identity: Ilya Rybak, Jorge Golowasch

This study has been carefully reviewed by two experts and the major criticism is summarized below. The main focus of the presented work is the question of 'What constitutes robustness of activity in systems in which the ionic conductances show high levels of variability?' By addressing this question the article does advance the field. Despite this positive comment both reviewers formulated a detailed long list of major and minor criticisms which strikes the center of the study. In the following summary only the major critical points have been summarized. However, we strongly recommend that the authors address all points raised by the reviewers. (1) The main objectives and goals of this work and, finally, the obtained results in relation to these objectives and goals are not clearly formulated. (2) The model of Hill et al. (2001) is one of the best examples of computational neural models in neuroscience. In the present manuscript, the author analyzed the robustness and sensitivity of that model to variation of some key parameters. Please carefully address the following questions: What can this study add to that classical model? Does this study allow a new evaluation of this model and/or suggest how to improve it, or can it confirm that the original model is good enough? Explicitly discuss the significance of the results in the Discussion and Significance Statement. (3) Please provide in the discussion the implications of the new discoveries. (4) Please improve the writing style of the manuscript. In particular remove all typos, grammatical and syntactical errors.

Reviewer 1:

Major points

I believe that the manuscript may be significantly improved by focusing on the following issues:

1. I would advice the authors to clearly formulate the main objectives/goals of this work and, finally, the obtained results in relation to these objectives/goals. It is not completely clear from the paper (as it is written) whether the authors (a) present a new method of parameter search in a large parameter space based on a novel method allowing a reduction of this space, which can be applied to many other models, but is currently applied to a particular, previously published model as an example, or (b) they specifically focus on the analysis of the robustness of this particular model to the variation of some of its key parameters.

So it would be very useful for a reader if the authors could clearly state their objectives/ goals from very beginning, like "The objective(s) of this study was (were) ....", in the Abstract and Introduction and then provide discussion the obtained results in the Discussion in relation to the formulated objectives. This does not require significant changes in the text, but adding clearly formulated statements in the Introduction and Discussion and a better formulation of these issues in the Abstract and Significance Statement.

In the former case (a), some general conclusions about the proposed search method and its advantages should be formulated and discussed independent of the particular model used, so that the researchers could understand how to use this novel approach/method in the analysis of other models.

In the latter case (b), it would be interesting to see some discussion concerning the range of the selected parameters from the literature, measured from real biological preparations, and the anticipated effects of their changes (e.g. by some pharmacological blockers) on the generated rhythmic activity, such as the period of oscillation (if such data exists), or to see the corresponding testable predictions (if such data does not exist).

The model of Hill et al. (2001) is one of the best examples of computational neural models in neuroscience. In the present paper, the author analyzed the robustness/sensitivity of that model to variation of some key parameters. So the question is what to do with this new knowledge. What this study can add to that classical model? Does this study allow a new evaluation of this model and/or suggest how to improve it, or can it confirm that the original model is good enough? I would advice the authors to explicitly discuss the significance of their results (what can be done based on these results or how they can be used) in the Discussion and Significance Statement.

2. Many parameters that define the kinetics of a particular ionic channel can affect the value of the corresponding ionic current and the performance of the entire model. For example an ionic current can be changed by changing the maximal conductance, or without changing the maximal conductance, by changing some other channel parameters, such as half-activation or half-inactivation voltages, slopes of voltage-dependent characteristics, and so on. In this case, the selection of parameters to be included in the space of parameters for search should be somehow justified. The authors stated that "...neuronal networks of animals are robust to natural variations, modulation and environmental perturbations of parameters like maximal conductance of intrinsic membrane and synaptic currents. Our study here focuses on the role of all these maximal conductances ....." However, there is a difference between using parameters "like maximal conductances" and using only maximal conductances. The use of maximal conductance as the only unknown parameter of a voltage-dependent channel could be justified as soon as all other voltage- and time- dependent characteristics of this channel have been experimentally measured from a representative neuron type. If the values of all these parameters were only suggested, but not measured, then what justification can be used for including in the parameter space only maximal conductances and keeping all other unknown channel parameters fixed. This issue should be discussed in the Discussion.

Minor

Abstract:

How a reader can understand from the Abstract the difference between the "realistic HCO" and the "functional "HCO", and what is "h" and "P"? In my opinion, all non-standard terms in the Abstract should be either defined or removed.

Line 11: please clarify and rewrite: "when working individually in the robust maintenance of normal alternating bursting activity"

Line 13: replace "CPG (central pattern generator)" with "central pattern generator (CPG)"

Line 20: change "families that have a biological meaning" to families that varied only by one parameter while the remaining parameters were held constant (or something equivalent).

Lines 24-28: The statement " Our results showed that the HCO system is very robust to variation in h which in turn consistently and substantially modulated period. The system is very sensitive to variations in P but functional bursting is stabilized by synaptic inhibition. We also found that period is determined by the interactions of shifting sets of parameters across the parameter space, but is easily and predictably modulated by varying h" is very difficult to understand. I would advice the authors to formulate their main results clearly and more general, so that people could understand their main achievements by reading the Abstract?

Significance Statement:

I would advice to include a more generally formulated significance of the work without details concerning concrete currents.

Results and Discussion

Line 99: replace "our own" with "new" or "novel"

Line 100: "instances that have a biological meaning" not clear what this means, I would suggest removing this and replacing it with a short explanation of a family.

Line 191: please clarify "and all the g's within the same family form the family's sequence"

Line 195: according to your definition "realistic bursters" should be a subgroup of "bursters", however, the numbers given here don't correspond to that.

Line 289: please explain "As a statistic"

Line 293: "this" to "the"

Line 306: change "Using this criterion, from above," to "Using these criteria"

Line 314: replace "(starting and ending gh values)" with something like "sorted by their starting and ending g values". Throughout the text you might even consider changing "starting" and "ending" to "lowest" and "highest" since especially with interrupted families there is not really a "start" or "end".

Line 419: change "if enough CaS is present" to "if gCaS is sufficiently high" (or similar)

Line 423: change "model neurons had enough CaS" to "model neurons had sufficiently high gCaS" (or similar).

Line 732: remove "biological meaning" and add a short explanation of "family"

Figure legend 3 and 4: you use the term "circuit break" that is not defined anywhere else, please make consistent with the rest of the text

Figure 1:

- please add the unit of Eleak in the key and I suggest giving Eleak values in mV instead of V

also correct the last one Eleak = -0.5, should be -0.05 V or -50 mV

Figure 8:

- I suggest rearranging the panels from high to low sensitivity (or vice versa)

- Please remove the grey line at 100% and the stars since they don't add to the understanding, 100% is per definition the canonical model and since this model is not represented in the figures as you describe in the text, indicating it is not just unnecessary but also confusing. The stars are not mentioned in the figure legend and don't serve a purpose, as far as I can tell.

Reviewer 2:

Neurons have been recently shown to express well-defined activity (e.g. bursting, tonic firing) in spite of the fact that their ionic conductance levels can vary over very broad ranges. Correlated expression of these conductances has been claimed to be important to maintain activity more or less bounded as conductances vary. This is best studied and has been confirmed in systems in which neuronal cell types can be identified without ambiguity, such as small invertebrate and vertebrate networks. The present work addresses the question of what constitutes robustness of activity in systems in which the ionic conductances show such high levels of variability and makes some important points. In that way, the article does advance the field. For this the authors use computational models of rhythmically active networks previously identified in leech heartbeat CPGs. By building millions of networks made of a pair of reciprocally inhibiting neurons (half-center oscillators, HCOs) that only differ in the values of the maximal conductances of the two cells, the authors examine how difficult it is to disrupt rhythmic activity in these HCOs as each of the 9 maximum conductances that make up these cells is varied over large ranges of values.

Major critiques

1) The authors identify ionic currents that the rhythmic activity is sensitive to and ionic currents that it is not (i.e. that the activity is robust to). This study is important as it identifies currents that are likely to be primary targets for neuromodulation in this system and those that the system is either sensitive or robust to. However, it does not, in my opinion, go nearly far enough in discussing the implications of these discoveries. In fact, I find the discussion of this manuscript to be mostly a long summary summary of the results with little other discussion of the implications of their study. Questions that one would like to see addressed, for example, might be: are the currents identified in this study as most likely to be modulated actually modulated in the biological system that inspired this work? Ih modulation in cortex is mentioned, but is the system a reasonable model of cortical networks where this occurs? Are the currents that the authors identify here as those that the system is robust to the ones that the biological system allows variations of? Since this study is not particularly novel in its approach (but certainly much more comprehensive than previous ones), it is not entirely clear what the fundamental message is other than assigning approximate scores of robustness of the system to each ionic conductance modified, and stating that IH is a good target for modulation (and this is a valid statement only if modulation is acceptable as long as loss of rhythmic activity is not the result). However, modulation may be necessary to switch activity modes.

2) Although the manuscript is pitched as a study of robustness in the context of ionic current correlated expression (which was more thoroughly address in a previous paper from this group), the authors begin their description of the system with a figure that describes a (non-linear) correlation between 4 parameters in the system (Fig. 1) but never return to address any possible significance of this. Also, the issue of correlations seems to be forgotten after that, while I would have wished to read something about the existence or lack of existence (unless I missed it) of correlations within the subfamilies they identify.

3) The significance statement really isn't a significance statement but another summary.

4) Additionally, this manuscript appears to be carelessly written and that makes it sometimes difficult and annoying to read: there are many typos and grammatical or syntactical errors that are too numerous to list here, but that should be heavily revised; another example is the presence of a figure in the first page of the manuscript (after the title page) that has no number, no description and is not referred to in the text anywhere; yet another example is the absence of the references to Calabrese, 1997 and Talebi &amp; Baker, 2012, in the bibliography. Other confusing/unclear aspects of the manuscript are listed below.

Minor critiques

1) Abbreviations in the abstract appear without explanation (e.g. h in line 24, P in line 25)

2) Line 50: Talebi &amp; Baker, 2012 reference is missing

3) Line 86: compartment should be "cell" really

4) Lines 143-145 and also legend in Fig. 8 (and panels B and C in that figure). This "mapping" of Eleak values to a % is confusing and completely unnecessary. In fact, you don't do that in Fig. 1. Why not move the Eleak points to the right side of each panel and add proper membrane potential labels to the right axis?

5) Line 163. Actually spiking frequency should be number of spike intervals (or spikes-1) divided by burst duration.

6) Line 288-289. The definition of continuous (non-interrupted) families is unclear to me. Here they are described as "each member has a unique value of g (that defines it), and these gs form a continuous uninterrupted sequence." I initially thought that uninterrupted families would be cases were families are missing for intermediate values of the defining conductance within the full 0-175% conductance range (e.g. the case of GCaS=[25,150] in Fig. 4). But "interrupted" appears to really mean a smaller range than the tested range (i.e. truncated). Still, it seems strange that for a given conductance only the families with the most members in it have uninterrupted families (Figs. 3 and 4). I guess that I both do not understand the definition well enough, and that the implications are also not clear. In fact, I find subsections starting in line 312 on "sequence break down" current by current tedious and not terribly informative. Perhaps a better description, ideally in some clever graphical form, and later discussion of the underlying implications would be more interesting.

7) Lines 318-334. This section is very confusing to me. There are statements that are simply not consistent with what the referred figure (Fig. 3) shows. For example, in line 328-329 it is said that "as the sequences' ending gh values were increasing ... the number of continuous families decreased and the number of interrupted families increased." What I see across columns (for any one row except the 3rd) that the continuous families decrease but always increase again at the highest ending gh (i.e. they are not monotonic). Also, it would be much easier to follow the description of numbers of families as gs vary if these numbers were actually shown on the subplots. I would suggest that in both Figs 3 and 4 the bracketed conductance values [g_start,g_end] were replaced with the # of families and that the entire graph was given labels at the margins to indicate the starting (vertical dimension) and ending g (horizontal dimension).

8) Lines 331-333. "In conclusion, the sequences broke down at low starting gh, but not at high starting gh (175%, 150%) where we found the most continuous families". That is also not what I see. In fact, at the limit of the smallest range of conductances (first subpanel in each row) is where we find most continuous families (indeed, all are continuous).

9) Line 386. The title does not reflect the text that follows. This and the following section (line 401 should be collapsed into one.

10) Section starting at line 401. This refers to Fig. 5, which I also find extremely confusing. The y-axes are labeled "Nr of missing members" but each plot has a header that also refers to missing members, yet these are 2 orders of magnitude different, and most certainly do not refer to the same thing. Then, what is "one missing member" or "7 missing members"? The text talks abot families with x members and not about families with x missing members like the figure describes.

11) Line 602. The conductance should be GsynS instead of GsysG, right?

12) Lines 618-634. This seems to me that should be in Methods and seems rather a trivial fitting and derivation procedure. What am I missing? Also, why use a 5th order polynomial for the fits when the curves look like simple exponentials?

13) Line 633. The threshold values 2.5 and 3 mentioned here appear to be slope values. Yet I can't see where a slope of 2 comes from. Also how can the low sensitivity group have a higher slope than the high sensitivity one?

14) The description in the text of Fig. 8 is again quite confusing. The figure shows panels for 8 member families (B) and 4 member families (C). First, the y-axes should be labeled. Second, the text instead describes numbers of families for 6-8 members, 3-5 members, less than 7, etc., which cross the borders of what the figures show and are in general not consistent with the figure's data.

15) Lines 704 and 706. The use of the description that conductances "affect each other" is not appropriate here. They are really not affecting each other. They are affecting activity, or they are related (or correlated?) to each other, but they do not affect each other.

16) Line 707. gK2 and gP are said to compensate for each other. This does not seem obvious to me as both gK2 and gP change non-monotonically, and thus direct relationships are not obvious. Are there any correlated relationships for any of these sensitivity subgroups perhaps? If they compensate for each other, I would expect to see a positive (linear or non-linear) correlation. Is that the case?

17) Lines 739-740. The lack of correlations mentioned here refers to the entire dataset, correct? Again I wonder if there may be any correlations (linear or not) between currents in each of the sensitivity subsets.

18) Line 787. "out of the physiological range" seems to me better than "out of the animal range".

19) Line 880. "Eleak has the same role as K2". I can't see this. An increase in Eleak (current becomes increasingly inward) is compensated for by an increase in K2 (outward current increases). If anything, they have opposite roles.

20) Line 890. "Mediums"?

21) Line 891. What is w.r.t.?

22) Lines 916-917. I like this conclusion. It is intuitively right but still a nice and clear point to make.

23) Line 927: Calabrese 1977 reference is missing

24) Line 1031. "All subpanels" Do you mean panels or bins?

25) Line 1034. Why "Circuit break"? What breaks here is the existence of families with certain member size, not the circuits.

26) Legend Fig. 8. It would be nice to show a scale bar for the circle diameters. I am not sure who is scaled x5 and who isn't.

27) Table 1. Top row of numbers should be labeled "# of members" or something like that.

28) Line 1079. "with all members having..." Do you mean members or families?

29) Fig. 6. Shouldn't Y-axis say "nr of missing families" instead?
